# A MYB-related transcription factor from sheepgrass, *LcMYB2*, promotes seed germination and root growth under drought stress

**DOI:** 10.1186/s12870-019-2159-2

**Published:** 2019-12-18

**Authors:** Pincang Zhao, Shenglin Hou, Xiufang Guo, Junting Jia, Weiguang Yang, Zhujiang Liu, Shuangyan Chen, Xiaoxia Li, Dongmei Qi, Gongshe Liu, Liqin Cheng

**Affiliations:** 1 Key Laboratory of Plant Resources, Institute of Botany, The Chinese Academy of Sciences, Beijing, China; 2 College of Management Science And Engineering, Hebei University of Economics and Business, Shijiazhuang, China; 3 Institute of Millet Crops, Hebei Academy of Agricultural & Forestry Sciences, Shijiazhuang, China; 4 Branch of Animal Husbandry and Veterinary of Heilongjiang Academy of Agricultural Sciences, Qiqihar, China; 5 Agro-Biological Gene Research Center, Guangdong Academy of Agricultural Sciences, Guangzhou, China

**Keywords:** *LcMYB2*, Seed germination, Root growth, Osmoprotectant

## Abstract

**Background:**

Drought is one of the most serious factors limiting plant growth and production. Sheepgrass can adapt well to various adverse conditions, including drought. However, during germination, sheepgrass young seedlings are sensitive to these adverse conditions. Therefore, the adaptability of seedlings is very important for plant survival, especially in plants that inhabit grasslands or the construction of artificial grassland.

**Results:**

In this study, we found a sheepgrass MYB-related transcription factor, *LcMYB2* that is up-regulated by drought stress and returns to a basal level after rewatering. The expression of *LcMYB2* was mainly induced by osmotic stress and was localized to the nucleus. Furthermore, we demonstrate that *LcMYB2* promoted seed germination and root growth under drought and ABA treatments. Additionally, we confirmed that LcMYB2 can regulate *LcDREB2* expression in sheepgrass by binding to its promoter, and it activates the expression of the osmotic stress marker genes *AtDREB2A*, *AtLEA14* and *AtP5CS1* by directly binding to their promoters in transgenic Arabidopsis.

**Conclusions:**

Based on these results, we propose that *LcMYB2* improves plant drought stress tolerance by increasing the accumulation of osmoprotectants and promoting root growth. Therefore, *LcMYB2* plays pivotal roles in plant responses to drought stress and is an important candidate for genetic manipulation to create drought-resistant crops, especially during seed germination.

## Background

Seed germination and seedling establishment are the most critical stages in the life cycle of plants [1], also the germination and young seedlings establishment phases for most plants are the most sensitive to stressful conditions, such as drought. Drought has been known as the most important factor limiting plant growth and productivity, particularly in arid and semiarid regions. Thus, the research on seed germination and seedling establishment under drought stress is required not only for evaluating and breeding the ecological adaptation of the species, but also for developing effective strategies for restoration and application arid and semiarid grassland.

Drought typically has complex ramifications that impose hyperosmotic and ionic stresses on cells by disrupting the water potential gradient of the soil-root-plant-atmosphere continuum [2]. Plants, especially those constantly growing in severe environments, have evolved many strategies to cope with abiotic stress. Generally, plants adopt either stress tolerance or stress avoidance strategies. For example, establishing deeper roots, which helps roots absorb water from deeper underground in drought conditions, is an avoidance strategy [2, 3]. Controlling stomata aperture to reduce transpiration and accumulating osmoprotectants (e.g. proline or soluble sugars) are usually thought to be stress tolerance strategies [4–7].

The primary signal caused by drought is osmotic stress, which is a signal that overlaps with that of salt stress and increases the accumulation of phytohormone abscisic acid (ABA) [8, 9]. Endogenous ABA plays a critical role in regulating stomatal aperture and inducing the biosynthesis of osmolytes through signaling cascades [10–13]. ABA increases, it binds to soluble ABA receptors (PYR/PYL/RCAR), and together they bind to and inhibit plant protein phosphatases (PP2Cs) [14–17]. The inactive PP2Cs are released from SnRK2 kinases, activating them so that they phosphorylate and activate downstream transcription factors and effectors in ABA response pathways [2, 18, 19].

Many types of transcription factors, such as basic Leucine Zipper (bZIP); NAM, ATAF and CUC transcription factor (NAC); APETUAP2/Ethylene-Responsive-Element Binding Protein (AP2/ERF), and MYB DNA-binding domain protein (MYB), are involved in drought stress responses [20–22]. MYB transcription factors are reported to play a role in multiple functions, including metabolism, cell fate and identity, developmental processes and responses to biotic and abiotic stresses during the plant life cycle [23]. Members of the MYB transcription factor family are divided into four classes depending on the number of adjacent repeats (one, two, three or four) [23]. It has been reported that *AtMYB60* and *AtMYB61* regulate stomatal movement and plant drought tolerance in opposite manners [24, 25]. *MYB96*, which is involved in the development of lateral roots, regulates drought response by integrating ABA and auxin signals [26]. In addition, the overexpression of *AtMYB44*, *StMYB1R-1*, *TaMYB33* or *TaPIMP1* improved drought stress tolerance in transgenic plants through different mechanisms [27–30]. An important discovery, that a 366 bp-insertion including three **MYB** ***cis*-elements** in the promoter of ZmVPP1 confers drought-inducible expression to ZmVPP1 in the variant, indicates that MYB-type transcription factors have significant functions in drought response [31]. Sheepgrass, which is widely distributed in Eurasia, adapts well to drought, cold, saline and alkaline conditions [32, 33]. To explore the mechanism that underlies the abiotic stress tolerance of sheepgrass, several transcriptome analyses have been performed in the past few years [32–35], and several genes identified by transcriptome analyses have actually enhanced the abiotic stress tolerance of transgenic plants [36–42]. Although MYB transcription factors play pivotal roles in drought responses, there is still no report on MYB proteins from sheepgrass that elucidates their contributions to sheepgrass drought tolerance. In a previous study, we revealed that 15 MYB and MYB-related transcription factors that are involved in drought stress response [35]. A transcript (contig41859), named *Leymus chinensis* MYB DNA-binding domain protein 2 (*LcMYB2*) is largely induced by drought and was selected for further analysis. The transcript levels of *LcMYB2* were enhanced under manninol, salt, ABA and cold treatments. The results of subcellular localization of 35S-LcMYB2-GFP and the distribution of the β-galactosidase activity of GAL4-BD-LcMYB2 indicate that LcMYB2 localizes to the nucleus and activates the transcription of lacZ. Chromatin immunoprecipitation (CHIP) analysis using anti-LcMYB antibodies showed that LcMYB2 can bind to the promoters of *Leymus chinensis* Dehydration Responsive Element Binding Protein 2(*LcDREB2*), *Arabidopsis thaliana* Dehydration Responsive Element Binding Protein 2A(*AtDREB2A)*, *Arabidopsis thaliana* △1-Pyrroline-5-carboxylate synthetas (*AtP5CS1*) and *Arabidopsis thaliana* Late-embryogenesis-abundant protein (*AtLEA14*). Overexpressing *LcMYB2* in *A. thaliana* promotes seed germination and enhances root growth under osmotic and ABA treatment and further increases soluble sugar and proline content with 300 mmol/L mannitol treatment. In addition, transgenic seedlings performed better than wild-type under natural drought stress. Taken together, these results indicated that *LcMYB2* plays critical roles in the drought responses of sheepgrass through both avoidance and tolerance strategies. Furthermore, this work provides important information for understanding the intrinsic characteristics of sheepgrass drought tolerance and supplies an important candidate gene for improving drought stress tolerance with genetic engineering.

## Results

### *LcMYB2* expression pattern analysis

Based on 454 high throughput sequencing and expression profile analyses of sheepgrass under drought stress, we found 15 MYB and MYB-related transcription factors that were responsive to changes of water content in plant tissues [32, 35]. Contig41859, which was up-regulated by drought stress and named *LcMYB2*, was a MYB-related transcription factor with unknown function that attracted our attention (Additional file 1: S1).

**Fig. 1:**
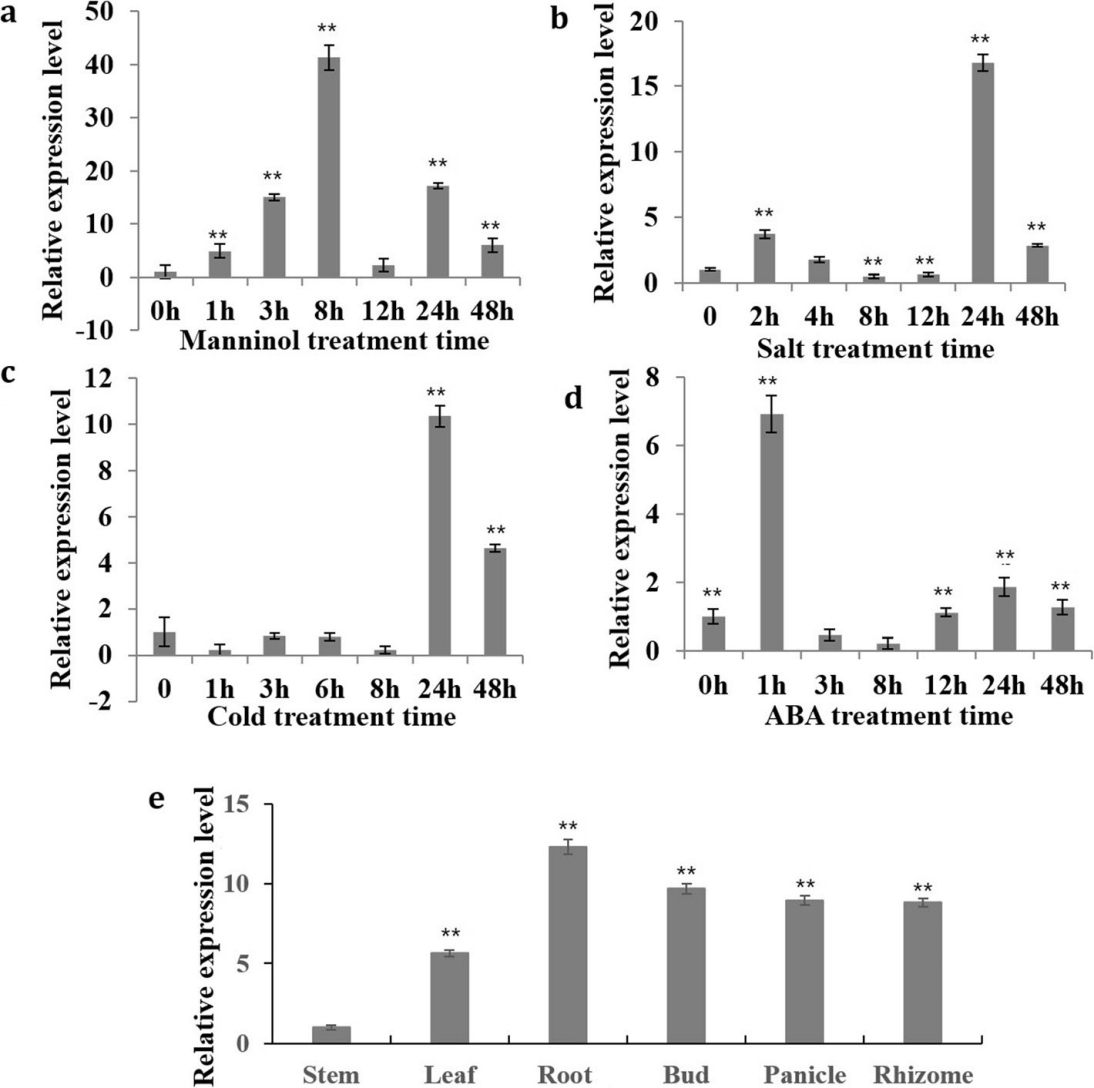
The expression patterns of *LcMYB2* under different treatments and its tissue-specific expression. **a** to **d** Expression of *LcMYB2* in the seedlings of 8-week-old sheepgrass were treated with or without 300 mM mannitol, 400 mmol/L NaCl, cold and 100 μmol/L ABA treatments for 0, 1, 3, 8, 12, 24 h or 48 h after stress treatments. **e** *LcMYB2* expression in stem, leaf, root, bud, panicle and rhizome of 1-year-old sheepgrass in flowering period. *LcACTIN* was used as a positive control for data normalization. Three independent replicates of measurements were performed for each time point, and the data are shown as the mean ± standard deviation (SD) (*n* = 3)

*LcMYB2* is highly induced by 300 mM mannitol at the 8th hour after treatment (Fig. 1a), whereas it is relatively slower responding to salt and cold stress (24 h, Fig. 1b, c). However, it is quickly upregulated by ABA treatment (Fig. 1d). The maximal level of mRNA accumulation under mannitol treatment is higher than under salt, cold and ABA treatments, indicating that *LcMYB2* mainly functions in response to osmotic stress. Furthermore, the expression level of *LcMYB2* in different organs is also detected under normal growth conditions. The results show that *LcMYB2* has the highest transcript level in roots (Fig. 1e). Based on these combined results, we predict that *LcMYB2* is mainly responsible for the osmotic stress response in roots, which may benefit plants under drought stress.

### Isolation and sequence analysis of the *LcMYB2*

**Fig. 2:**
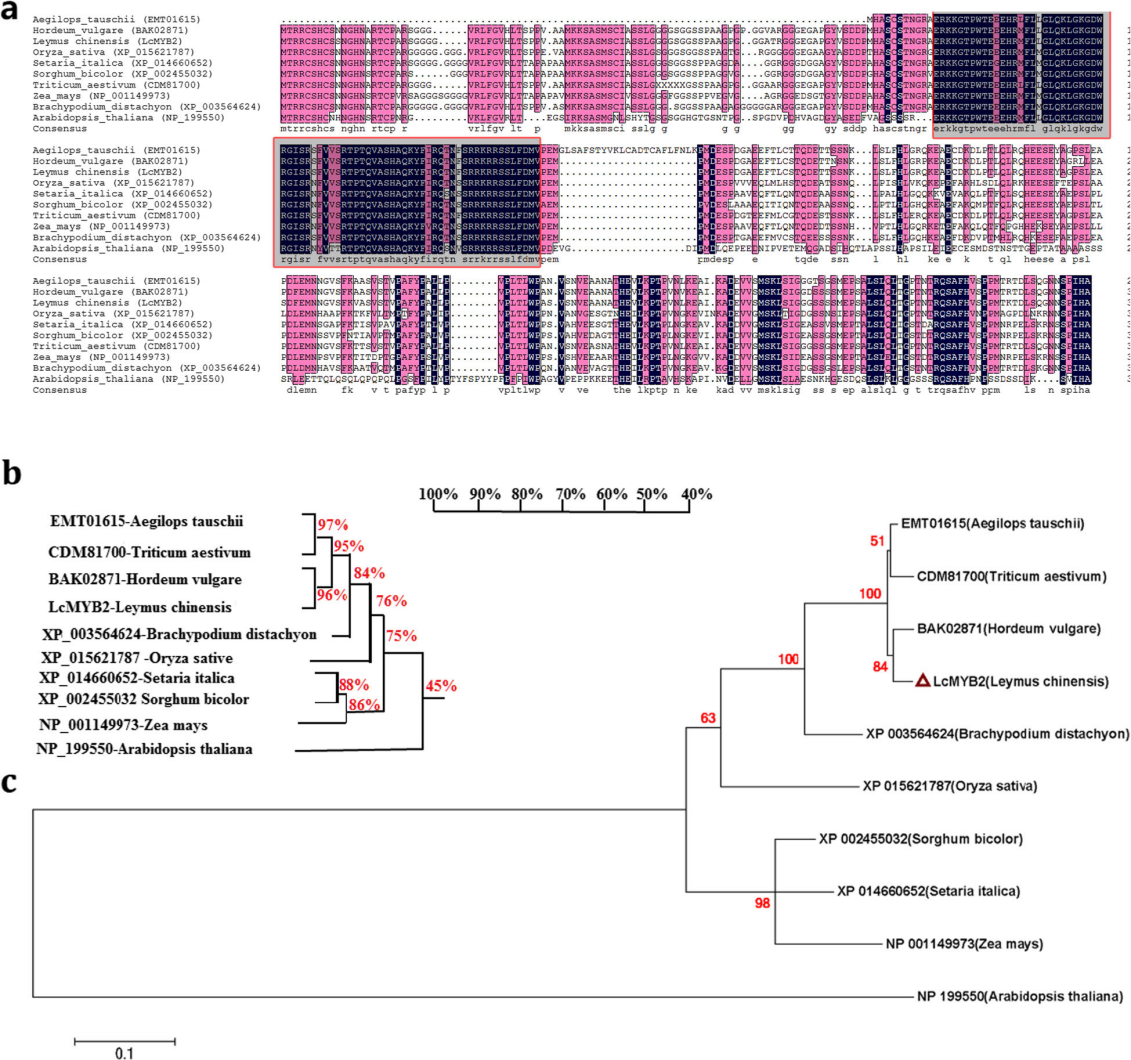
Multiple sequence alignment and phylogenic analysis of *LcMYB2* with homologous sequences on NCBI. **a** Sequence alignment using DNAMAN; **b** Homology tree constructed with DNAMAN. The numbers on the branches indicate the similarity of the sequences; **c** Molecular phylogenetic analysis by the maximum likelihood method with MEGA6.0; the numbers on the branches indicate the percentage of bootstrap support from 1000 replicates; the branch length represents the divergence distance

Putative full-length *LcMYB2* was isolated from sheepgrass by Rapid-Amplification of cDNA Ends-PCR (RACE-PCR) and classical PCR based on the 454 high-throughput data (SRA065691; Additional file 2: S2). The length of *LcMYB2* Open reading frame (ORF), region is 1092 bp, encoding 363 amino acids (GenBank: KY316376). The molecular mass of the putative protein is approximately 38.4 kDa, and its theoretical isoelectric point (pI) is 8.3 (predicted by DNAMAN 7.0). Multiple sequence alignment of *LcMYB2* with its homologs shows that a conserved domain exists among these sequences (amino acids 101–171; Fig. 2a). Sequence similarity and phylogenetic analysis show that *LcMYB2* forms a clade with BAK02871 (*Hordeum vulgare*), CDM81700 (*Triticum aestivum*) and EMT01615 (*Aegilops tauschii*) by a high nodal support values (Fig. 2b, c). However, the functions of these *LcMYB2* homologs have not been reported thus far. The analysis of *LcMYB2* biological function is of great significance for *Leymus chinensis* and closely related species *Triticum aestivum* and *Aegilops tauschii* homologs.

### Subcellular localization and transcription activity assay of *LcMYB2*

**Fig. 3:**
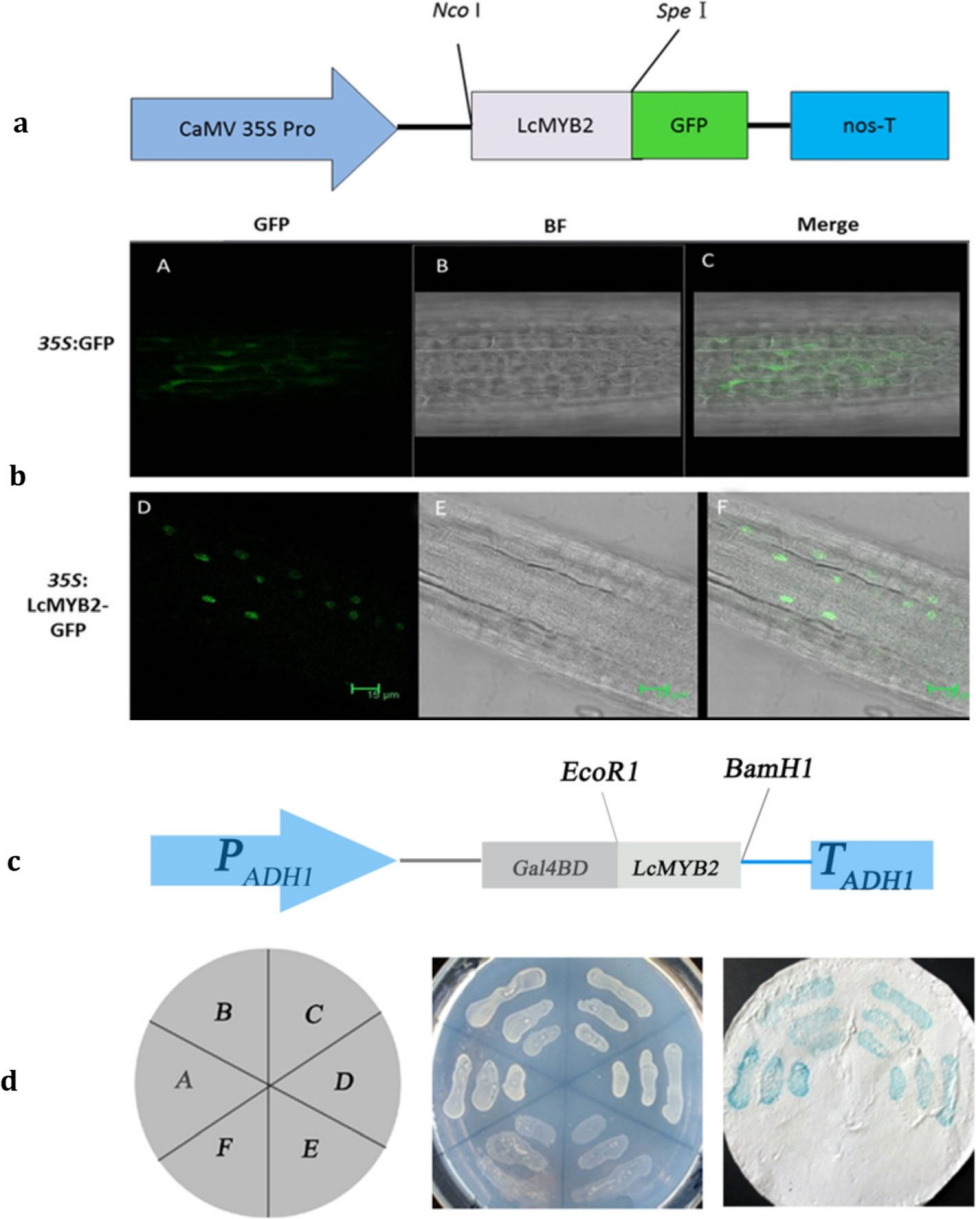
Subcellular localization and transcription activity of LcMYB2. (a) The LcMYB2-GFP construct; (b) Subcellular location of LcMYB2; A, D: GFP; B, E: bright field observation; C, F: merge; (c) The Gal4BD-LcMYB2 construct; (d) Transcription activity detected by X-gal staining, Left, The arrangement of yeast lines harboring different constructs, A and B are LcMYB2-containing yeast; C and D are positive controls; E and F are negative controls; Medium, The phenotype of yeast growing on SD/−His-Trp medium; Right, Beta-galactosidase activity assay of using X-gal

To determine the subcellular localization of *LcMYB2*, the ORF of *LcMYB2* (without the TGA stop codon) was fused to a GFP reporter gene under the control of the CaMV 35S promoter (Fig. 3a). Recombinant CaMV35S::LcMYB2-GFP and CaMV35S::GFP were transformed into Arabidopsis separately by the floral dip method. Confocal microscopy showed that the GFP protein was localized throughout the whole cell, whereas the LcMYB2-GFP fusion protein was present only in the nucleus (Fig. 3b), suggesting that LcMYB2 is a nuclear-localized protein.

The transcriptional activation of *LcMYB2* was tested using a yeast one-hybrid assay system. The *LcMYB2* ORF was inserted at the 3′-end of GAL-BD under the control of P_ADH1_ to form a BD-*LcMYB2* fusion gene (Fig. 3c). The yeast strain AH109, harboring BD-LcMYB2 or BD-WRKY15 (positive controls), grew normally on SD/−His-Trp medium, whereas AH109 harboring only BD (negative control) did not grow. In β-galactosidase activity assays on Whatman filter paper, blue signal appeared in the regions where BD-LcMYB2 or BD-WRKY15-containing yeast were growing (Fig. 3d). Therefore, we suggest that *LcMYB2* serves as a transcription activator and functions in the nucleus.

### Performance of transgenic plants under osmotic stress, ABA treatment and natural drought treatment

**Fig. 4:**
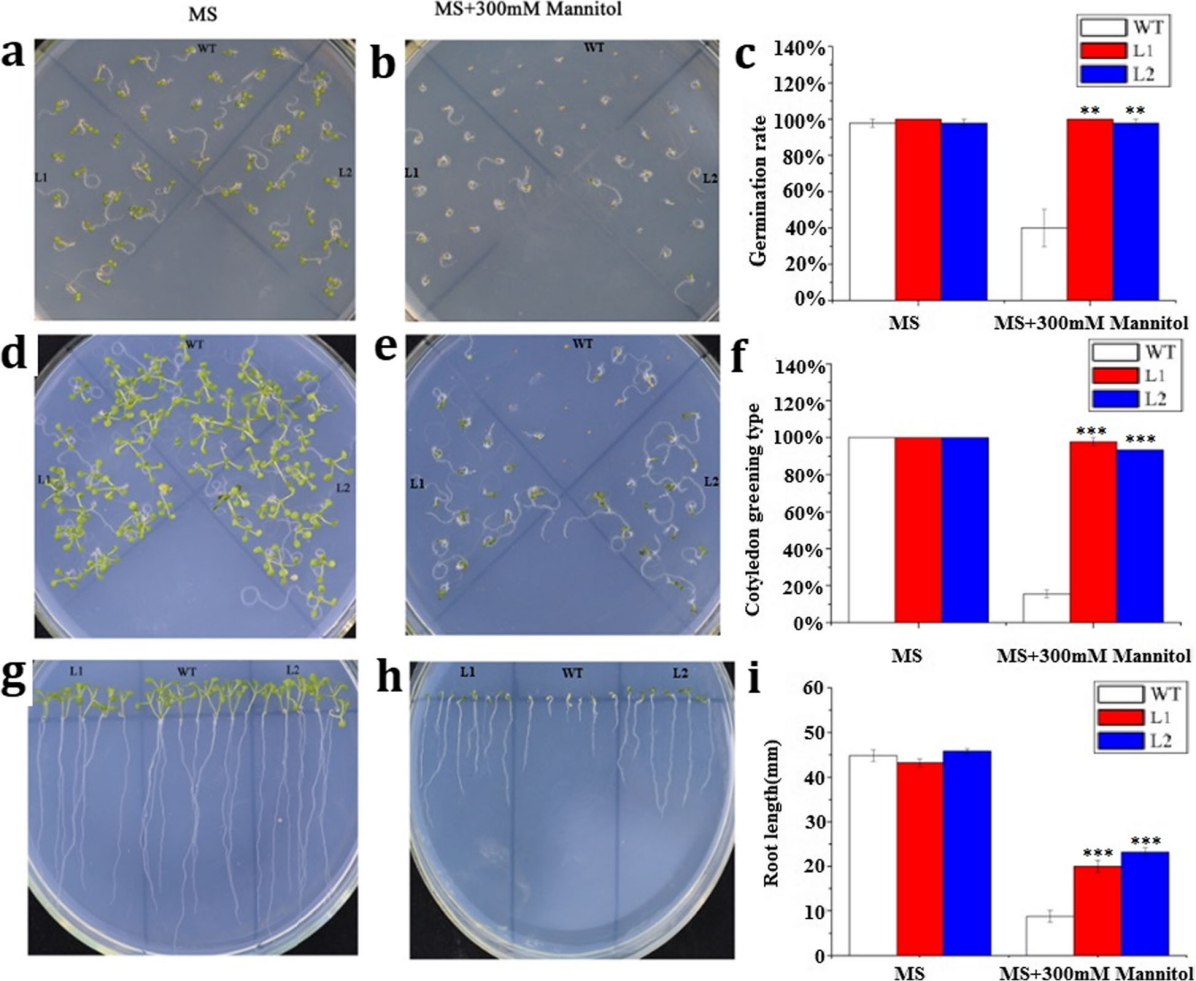
Phenotypic and statistics analysis of *LcMYB2* transgenic *Arabidopsis* under mannitol stress. **a** Germination on MS medium as control. Photos were taken on the 5th day; **b** Germination on MS containing 300 mM mannitol; **c** Statistics of germination rates; **d** Cotyledon greening rate on MS medium, Photos were taken on the 10th day; **e** Cotyledon greening rate on MS containing 300 mM mannitol; **f** Statistics of cotyledon greening; **g** Root growth on MS. Photos were taken on the 10th day; **h** Root growth on MS containing 300 mM mannitol; **i** Statistics of root length

**Fig. 5:**
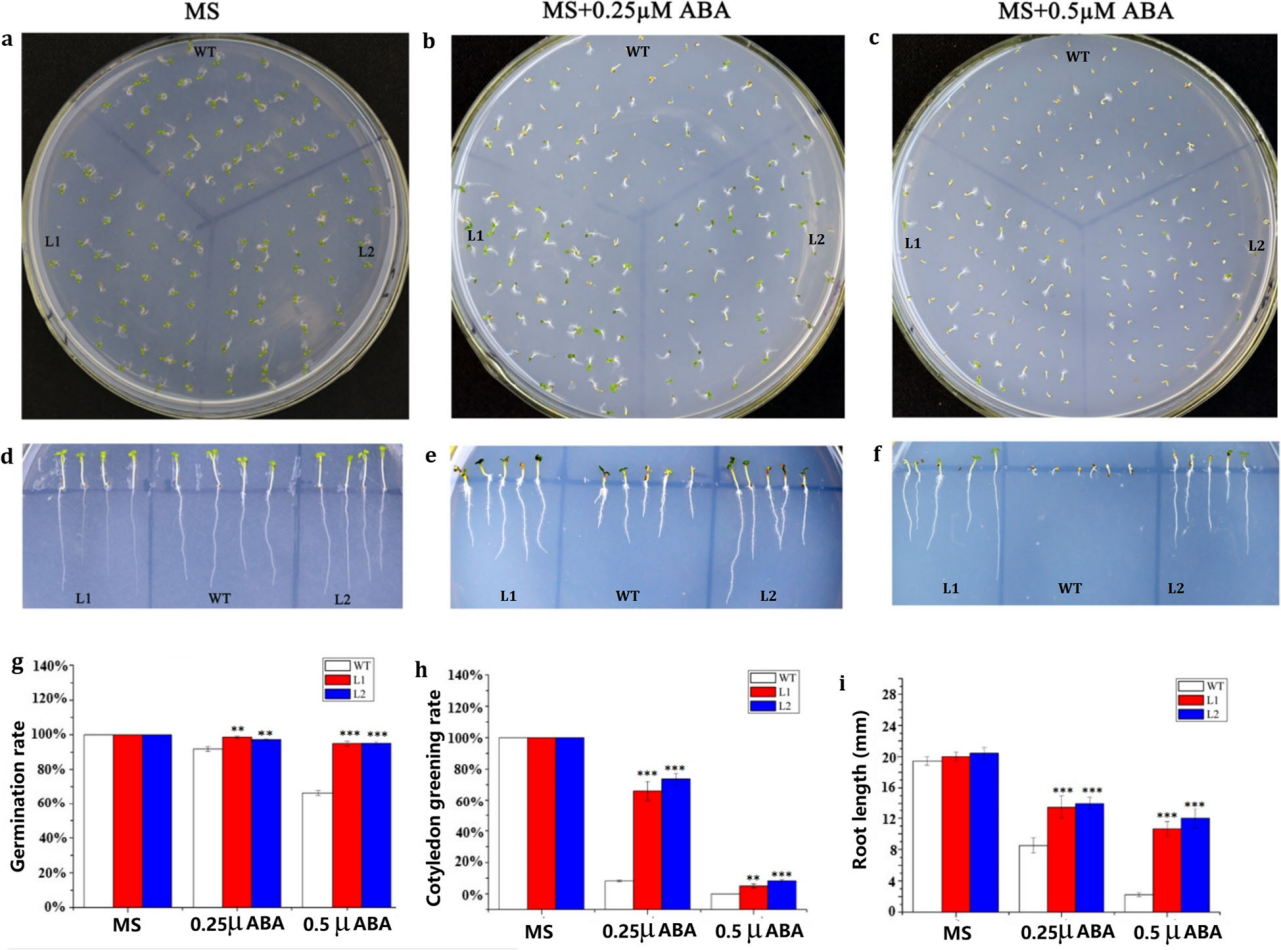
Phenotypic and statistics analysis of *LcMYB2* transgenic *Arabidopsis* under ABA stress. **a** Germination on MS medium as control; **b** Germination on MS containing 0.25 μM ABA; **c** Germination on MS containing 0.5 μM ABA; **d** Root length on MS medium; **e** Root length on MS containing 0.25 μM ABA; **f** Root length on MS containing 0.5 μM ABA; **g** Statistics of germination rates in different growth environments; **h** Statistics of cotyledon greening rates in different growth environments; **i** Statistics of root length in different growth environments. The germination and cotyledon greening rates were calculated halfway through the third day, and the photos of germination were taken on the 4th day after planting on medium. The photos of root growth were taken on the 5th day, and the root length was measured at the same time

**Fig. 6:**
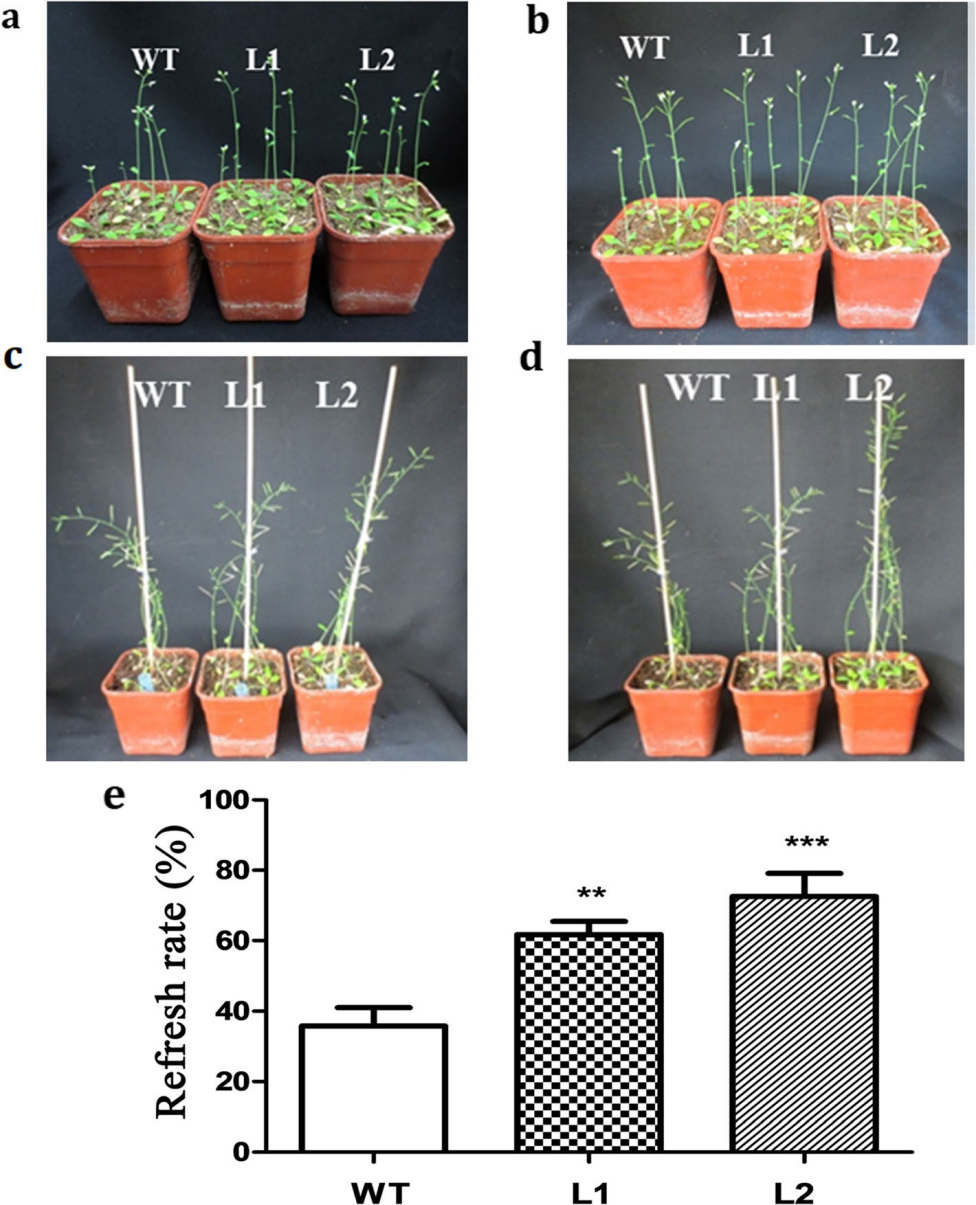
Phenotypic and statistics analysis of *LcMYB2* under natural drought. **a** Plants under natural drought stress for 21 days; **b** Plants under natural drought stress for 28 days; **c** Plants under natural drought stress for 42 days; **d** Plants 3 days after rewatering

First, we probed the biological functions of *LcMYB2* at the seed germination stage under osmotic or ABA treatment. Under normal conditions (Murashige-Skoog medium,MS), there were no significant differences between transgenic and wild-type seeds in germination rate, cotyledon greening rate or root length (Fig. 4a, d, g, c, f, i; Fig. 5a, d, g, h, i; Additional file 5: S5). Under treatment with 300 mmol/L mannitol, there were significant differences in germination rate (*p* < 0.01; Fig. 4b, c), and the cotyledon greening rate and root length had very significant differences (*p* < 0.001) between transgenic and wild-type seeds (Fig. 4e, f, h, i; Additional file 5: S5). Under treatment with 0.25 μmol/L ABA, the germination rate (*p* < 0.01), cotyledon greening rate (*p* < 0.001) and root length (p < 0.001) were significantly different between transgenic and wild-type seeds, and similar results were obtained with 0.5 μmol/L ABA treatment (Fig. 5b, c, e, f, g, h, i). Taken together, these data indicate that *LcMYB2* can promote seed germination and root growth under osmotic stress and possibly via the ABA signaling pathway. In addition, the transgenic plants maintained green leaves longer under natural drought stress conditions and had a higher refresh rate after rewatering than did wild-type (Fig. 6).

**Fig. 7:**
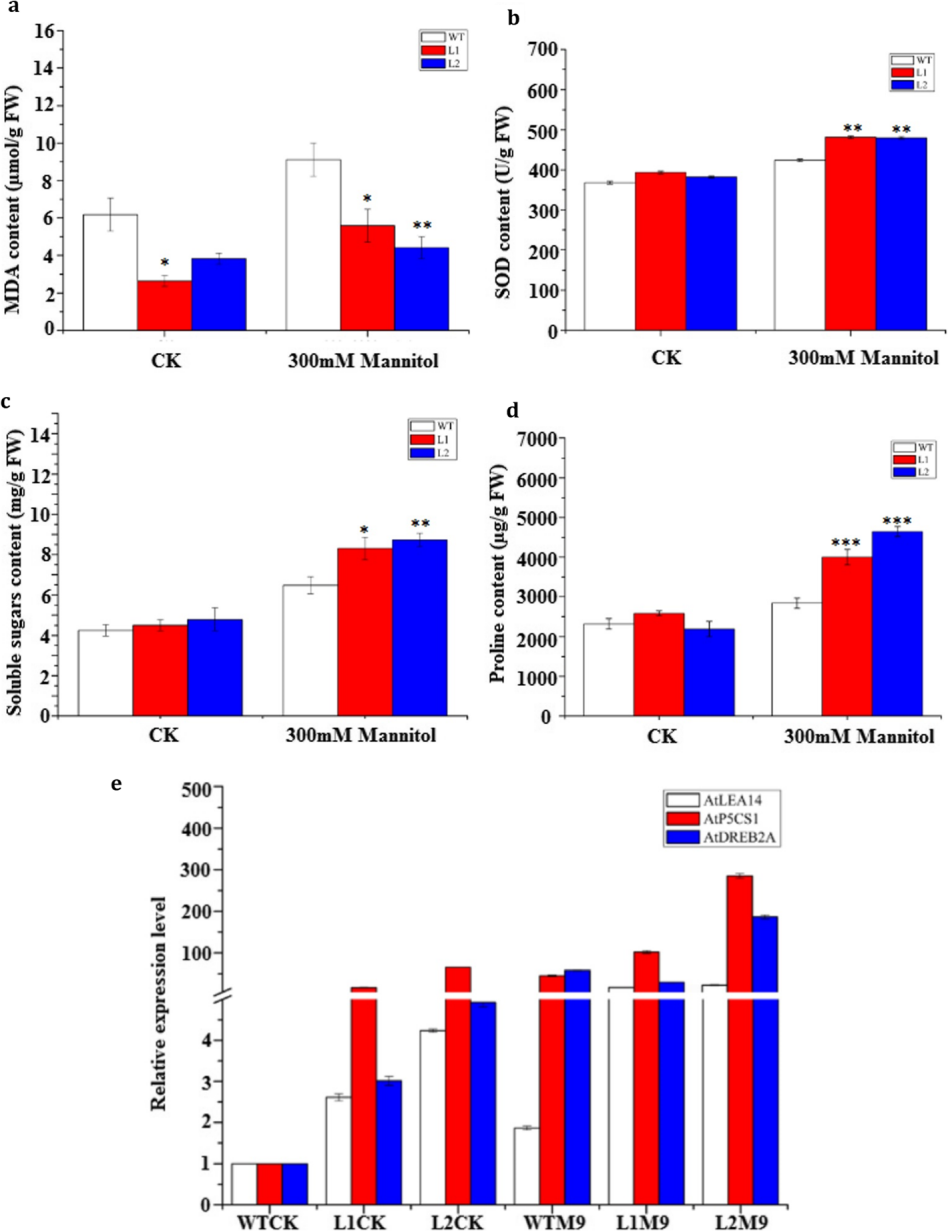
*LcMYB2* significantly increased the accumulation of proline under mannitol stress. **a** MDA content of wild-type and transgenic *Arabidopsis thaliana* under control conditions and osmotic stress; **b** SOD content of wild-type and transgenic *Arabidopsis thaliana* under control conditions and osmotic stress; **c** Soluble sugar content of wild-type and transgenic *Arabidopsis thaliana* under control conditions and osmotic stress; **d** Proline content of wild-type and transgenic *Arabidopsis thaliana* under control conditions and osmotic stress; **e** Changes in gene expression of *AtDREB2A*, *AtP5CS1* and *AtLEA14*. Physiological indices were measured with the wild-type and transgenic *Arabidopsis thaliana* exposed to 300 mM mannitol for 2 days

To investigate the physiological responses of transgenic and wild-type *A. thaliana* under osmotic stress, we irrigated 4-week-old seedlings with 300 mmol/L mannitol. Two days later, the malondialdehyde (MDA), Superoxide dismutase (SOD), soluble sugars and proline contents were measured. The results showed that the two transgenic lines overexpressing *LcMYB2* accumulated greater amounts of SOD (p < 0.01), soluble sugars (*p* < 0.05/0.01) and proline (p < 0.001) than wild-type lines under 300 mmol/L mannitol treatment, whereas they had lower MDA content (Fig. 7a, b, c, d). The greater accumulation of proline and soluble sugars in the transgenic lines might provide extra protection to the cells under drought stress. The lower MDA and higher SOD content indicates that less damage occurred in the cells of transgenic plants. These results together suggest that *LcMYB2* promotes osmotic stress resistance. The gene expression levels of *AtDREB2A*, *AtP5CS1*, and *AtLEA14* were measured by Quantitative Real Time PCR (qPCR) on the 9th hour after treatment with 300 mmol/L mannitol. The expression levels of these genes were higher in transgenic plants than in wild-type plants both in the control check (CK) and in the treatment group (M9; Fig. 7e).

### CHIP analysis

**Fig. 8:**
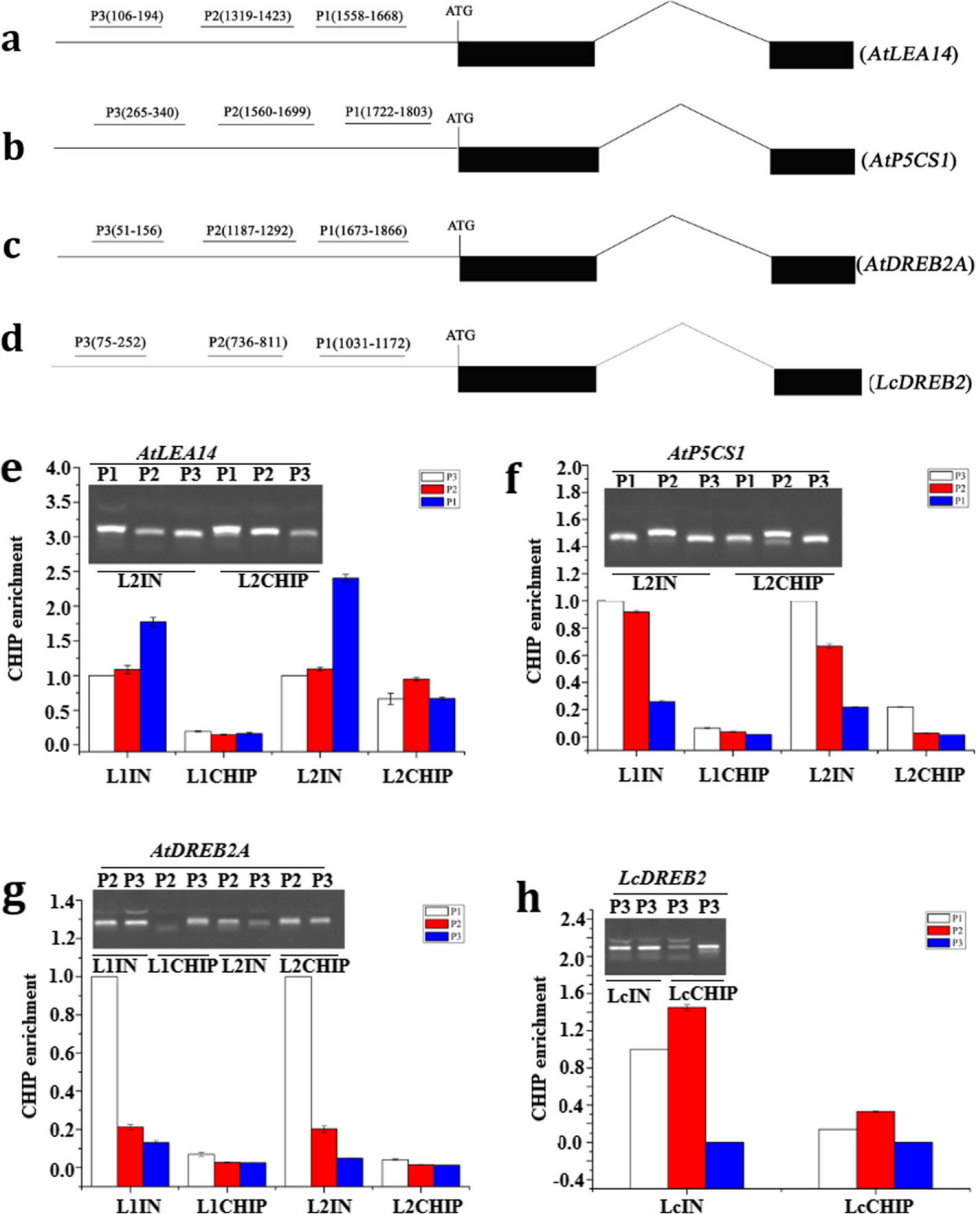
LcMYB2 binds to the promoter regions of targets, as revealed by CHIP. L1IN, L2IN, LcIN were the plant total nucleoproteins of transgenic *Arabidopsis* line 1, line 2 and *Leymus chinensis* leaf, while, L1CHIP, L2CHIP and LcCHIP were proteins that pulled down by antibody of LcMYB2 (anti-LcMYB2) from L1IN, L2IN, LcIN respectively. The DNAs released from these proteins were collected for PCR and qPCR analyses. **a** Schematic of the *AtLEA14* gene; **b** Schematic of the *AtP5CS1* gene; **c** Schematic of the *AtDREB2A* gene; **d** Schematic of the *LcDREB2* gene; **e** The binding of LcMYB2 to the promoter region of *AtLEA14.* **f**: The binding of LcMYB2 to the promoter region of *AtP5CS1*; **g** The binding of LcMYB2 to the promoter region of *AtDREB2A*; **h** The binding of LcMYB2 to the promoter region of *LcDREB2*

It has been shown that MYB proteins can recognize the motifs A/TAACCA and C/TAACG/TG [43]. Therefore, we analyzed the promoter sequences of *AtLEA14*, *AtP5CS1*, *AtDREB2A* and *LcDREB2* using the sequences ~ 1500–2000 bp upstream of the predicted transcription start sites (TSSs), and several possible motifs were found in the putative promoter regions (Additional file 3: S3). We further confirmed our prediction with CHIP experiments, and the signals were detected in qPCR and universal PCR reactions with DNAs, released from proteins LICHIP, L2CHIP or LcCHIP, as templates. These results indicated that the LcMYB2 (or together with its interaction proteins) regulates the expression of *AtLEA14*, *AtP5CS1*, *AtDREB2A* and *LcDREB2* (Fig. 8).

## Discussion

MYB and MYB-related transcription factors constitute a large family in plants and are involved in many biological processes, for example, secondary metabolism and responses to environmental factors [44–46]. Isolating and characterizing the functions of these genes provide a way to learn about plant-specific events at the transcriptional level. AtMYB2, a MYB-related protein, is induced by drought and by ABA; whereas BcMYB1 is rapidly and strongly induced by drought but only slightly by exogenous ABA, indicating that MYB proteins respond to environmental changes through both ABA-dependent and ABA-independent pathways [47–49]. *LcMYB2* is induced to its maximal level at 1 hour after treatment with exogenous ABA and 8 hours after treatment with exogenous mannitol (Fig. 1a, d). However, the extent of *LcMYB2* induction by mannitol is greater than by ABA, suggesting that *LcMYB2* functions mainly in an ABA-independent pathway.

When facing drought stress, plants always adopt avoidance or resistance strategies to mitigate the negative effects of the stress. Rooting deeply is one of the avoidance strategies. For example, the introduction of the DEEPER ROOTING 1 (DRO1) gene into a shallow-rooting rice cultivar increased the downward growth of roots, and the resulting transgenic lines had higher yields under drought conditions [3]. Our results demonstrate that *LcMYB2* is induced by mannitol and promotes root elongation at the germination stage and during seedling growth under osmotic stress and ABA treatment (Fig. 4 and Fig. 5). Therefore, *LcMYB2* has the potential to enhance plant root growth to avoid drought stress.

Osmotic adjustment is usually thought to be one of the main mechanisms of resistance to drought or salt stress. Compatible osmolytes, such as proline, soluble sugars and LEA proteins, are often measured as critical physiological criteria to evaluate the tolerance of plants to abiotic stresses. Proline biosynthesis is catalyzed by P5CS1/2 and P5CR, and it is thought to protect subcellular structures and macromolecules under osmotic stress [50, 51]. Petunias overexpressing *AtP5CS* or *OsP5CS* accumulate more proline and appear to have drought tolerance [52]. In addition, soluble sugars, especially sucrose or trehalose, are correlated with the acquisition of desiccation tolerance and are thought to stabilize the membrane structure in dry environments [53, 54]. LEA proteins, first found in cotton, were shown to be up-regulated by drought stress in many species and function as compatible solutes to maintain the cellular structure in severe dehydration conditions [54–56]. Overexpression of some LEA protein-encoding genes confers enhanced drought tolerance in transgenic plants [57, 58]. Here, we found that *LcMYB2* was induced by osmotic stress in sheepgrass (300mMmannitol); and *A. thaliana* overexpressing *LcMYB2* accumulated more soluble sugars and free proline and expressed higher levels of *AtLEA14*, *AtP5CS1* and *AtDREB2A* than wild-type *A. thaliana* seedlings under mannitol treatment (Fig. 7). In sheepgrass, many LEA protein-encoding genes and two P5CS-encoding genes were induced significantly by drought stress 35). Here, we demonstrate that LcMYB2 can bind to the promoter regions of *AtLEA14* and *AtP5CS1* (Fig. 8a, b, e, f). Therefore, we suggest that *LcMYB2* functions in sheepgrass by elevating the content of osmoprotectants.

DREB proteins have been extensively studied and have been shown to improve drought tolerance in transgenic plants. *AtDREB2A* and *AtDREB2B* are strongly induced by dehydration stress in roots and stems, and constitutive expression of *AtDREB2A* results in significant drought stress tolerance [21, 59]. In addition, *OsDREB2B* is markedly induced by various stresses, and overexpressing *OsDREB2B* in *A. thaliana* or rice increases the expression of *DREB2A* target genes and improves transgenic plant drought stress tolerance [60, 61]. Previous studies have proposed that DREB proteins, such as DREB1 and DREB2, regulate low-temperature and drought-responsive genes by binding to the DRE/CTR elements through ABA-independent pathway [62, 63].

In sheepgrass, the highest transcript level of *LcDREB2a* occurs at the 12th hour under 20% PEG6000 treatment [42], whereas *LcMYB2* transcript accumulation reaches the highest point at 8 h after 300 Mm mannitol treatment (Fig. 1a). Expression profile sequence analysis showed that both contig62249 (*LcDREB2C*/*LcDREB2B*/*LcDREB2A*) and contig41859 (*LcMYB2*) were up-regulated by drought stress and returned to basal levels after rewatering; however, the fold change of contig41859 was larger than that of contig62249 in response to drought stress (Additional file 1: S1). These results indicate that LcMYB2 is a possible transcription regulator upstream of *LcDREB2*. Therefore, we cloned the promoter sequence of *LcDREB2* (~ 1500 bp upstream of the predicted transcription start site, Additional file 2: S2) and assayed the binding of LcMYB2 protein to this promoter region using CHIP analysis. Universal PCR and qPCR enrichment of CHIP DNA revealed that LcMYB2 can bind to the promoter regions of both *AtDREB2A* and *LcDREB2* (Fig. 8c, d, g, h). Therefore, we propose that LcMYB2 improves drought tolerance by activating *LcDREB2* in sheepgrass. Also, we find that *LcMYB2* can bind to the promoters of sheepgrass *LcDREB2* and Arabidopsis *AtDREB2A*, maybe they all have MYB binding elements in their promoters, which shows that the mechanism of response to stress in plants is conservative. Otherwise, pearson correlation analysis with differentially expressed genes DEGs of sheepgrass transcriptome data under drought stress [35] to gain novel insights on the mechanism of LcMYB2 on drought stress, the pearson correlation analysis results showed that LcMYB2 expression level was perfectly positively correlated with the expressions of typical stress response genes of LcLEA, LcDREB2c, LcMYB39, Peroxidase 56 under drought stress in sheepgrass (Additional file 6: S6), which provided evidence for the analysis of LcMYB2 function under drought stress. The pearson correlation analysis results about the expression levels of LcMYB2 with LcLEA, LcDREB2c, LcMYB39 in sheepgrass were mutually underlying with the results of CHIP-PCR on LcMYB2 binding the corresponding promoter elements of AtLEA14, AtP5CS1, AtDREB2A and LcDREB2. This research integrates the RNA-seq further analysis, CHIP-PCR and universal PCR results, and provides the evidence for understanding the function of LcMYB2 under drought stress.

With the development of the sheepgrass industry (artificial cultivation, natural grassland improvement, and artificial grassland establishment), the drought resistance of sheepgrass during the seed germination and seedling establishment stages in water-deficient areas is critical for propagation and is necessary for reaping economic and ecological benefits. Therefore, *LcMYB2* is an important candidate for improving plant drought stress tolerance through genetic engineering.

## Conclusions

**Fig. 9:**
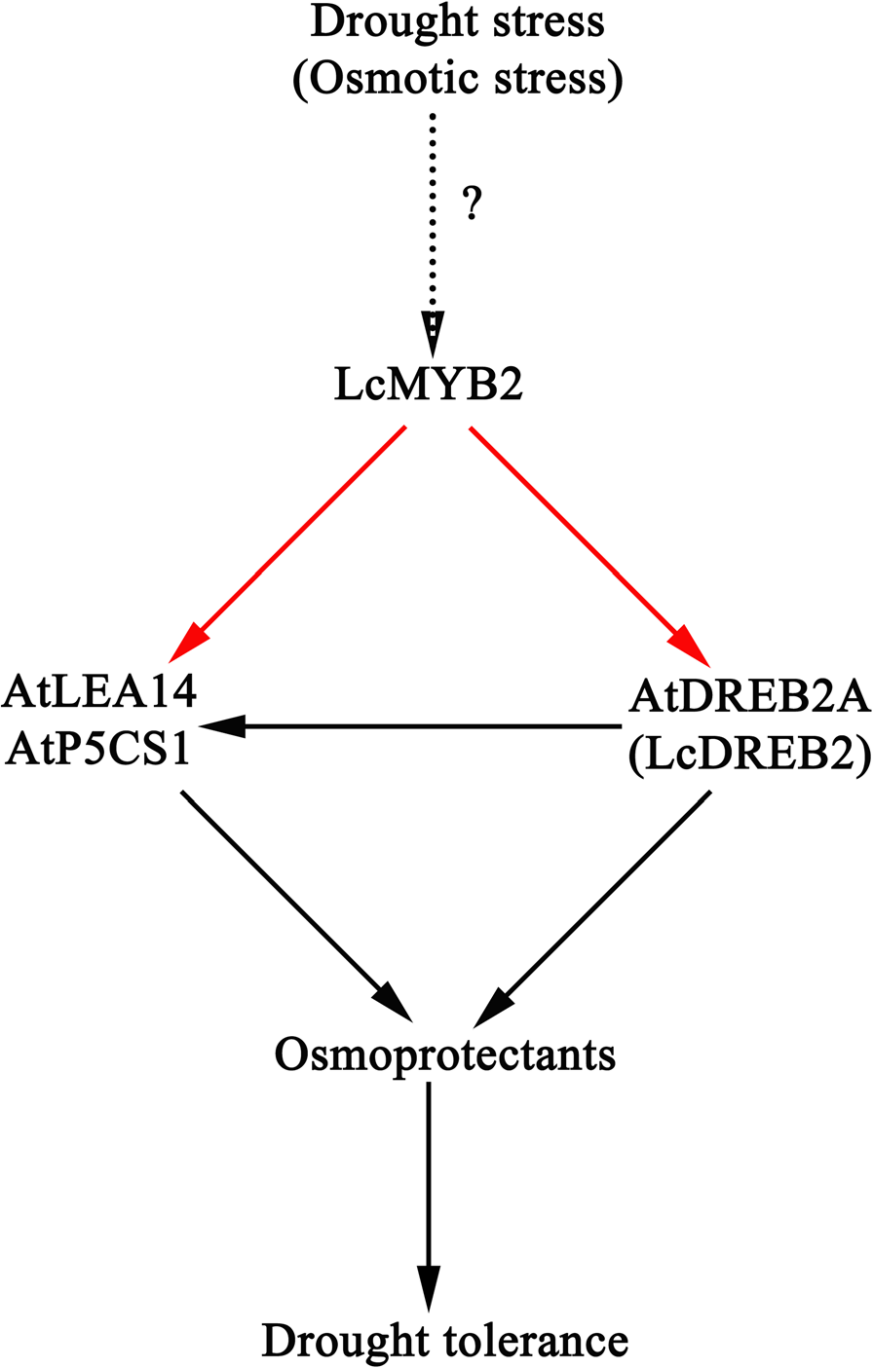
A hypothetical model of *LcMYB2* function in transgenic plants

In conclusion, we showed that a drought and osmotic-inducible transcription factor, LcMYB2, improves the drought and osmotic tolerance of plants by bingding the elements of promoters from the *AtDREB2*, *AtLEA14*, *AtP5CS1* and *LcDREB2* and regulating the transcription of drought-responsive genes to increase the accumulation of osmoprotectants (Fig. 9). These may be among the reasons underlying the tolerance of sheepgrass to drought-prone environments. Others, some the cis-elements of stress response genes from the monocotyledons and dicotyledonous plants are conserved in evolution, they both can be bound by the same trans-factors.

## Methods

### Plant materials and treatments

Sheepgrass (National certified variety Zhongke 1 from Institute of Botany, the Chinese Academy of Sciences, Beijing, China) was used for this experiment. The sheepgrass seedlings were grown in a plastic pot containing nutrition soil (Pindstrup Bubstrate, Denmark) and vermiculite (2:1, v/v) in the greenhouse at 27/23 °C, 16 h light/8 h dark for 8 weeks before treatments. Abiotic stresses were performed as follows: the plastic pots containing the seedlings were submerged in 100 μmol/L for ABA treatment, 300 mmol/L mannitol for osmotic stress and 400 mmol/L NaCl for salt stress. Seedlings were transferred to a growth chamber at 4 °C for cold stress. The seedlings were sampled at 0, 1, 3, 8, 12 or 24 h after stress treatments, immediately frozen in liquid nitrogen and stored at − 80 °C for RNA isolation. The abiotic stress experiments were excuted at least three times by different people to test the expression patterns of target genes. Stem, leaf, root, bud, panicle and rhizomes were also collected from 2-year-old sheepgrass seedlings for tissue-specific analysis.

*Arabidopsis thaliana* (*A. thaliana;* Columbia ecotype) seeds were surface-sterilized with 10% NaClO for 10 min, and then washed 5 times with sterile water. The sterilized seeds were planted on MS solid media (pH 5.8) for germination.

### RNA isolation and expression pattern analysis of *LcMYB2*

Total RNA was extracted using Trizol reagent (TaKaRa, Dalian, China) according to the manufacturer’s instructions. First-strand cDNA was synthesized using the PrimeScript™ RT reagent Kit (TaKaRa, Dalian, China) according to the manufacturer’s instructions. qRT-PCR was carried out in triplicate according to the SYBR PremixExTaq™ protocol (TaKaRa, Dalian, China) on a LightCycler480 Real-Time PCR System (Roche, Rotkreuz, Switzerland) with the following program: 95 °C for 5 s and 68 °C for 30 s for 45 cycles. Tissue-specific expression of *LcMYB2* was detected using semiquantitative PCR. All primers used in this research are listed in Additional file 4: S4.

### Amplification and sequence analysis of *LcMYB2*

First-strand cDNA for amplification of the 5′ and 3′ ends of *LcMYB2* was synthesized with SMARTer™ RACE cDNA Amplification Kit (Clontech, Palo Alto, CA) according to the manufacturer’s instructions. The gene-specific primer (GSP-5’RACE), designed according to the 454 high-throughput sequencing results, and universal primers (UPM) were used to amplify the 5′ end of *LcMYB2*. The putative full-length sequence of *LcMYB2* was amplified using gene-specific primers (LcMYB2-F/R) designed according to the sequence assembled from the 5′-RACE and 454 sequencing results. The national center for biotechnology information (NCBI) database was searched for homologs of *LcMYB2* using the BLASTX program. Multiple sequence alignment was executed in DNAMAN software (version 7.0) using the selected amino acid sequences. The phylogenetic relationships among the homologs were inferred using the maximum likelihood method based on the JTT matrix-based model in MEGA software version 6.0 [64, 65].

### Subcellular localization and transcriptional activity assay of LcMYB2

The ORF of *LcMYB2* was ligated into pCAMBIA1302 to form the *LcMYB2*-GFP fusion protein. The recombinant plasmid was introduced into *Agrobacterium tumefaciens* EHA105 using a freeze-thaw method and further transformed into *A. thaliana* using the floral dip method [66]. Positive seedlings were selected on solid MS containing 50 μg/L hygromycin (Roche) and further confirmed by PCR. T3 seeds of the transgenic plants were germinated on MS, and the GFP fluorescence in the roots was observed under a laser confocal scanning microscope (Leica TCS SP5). To assess *LcMYB2* transcription activity, the ORF was inserted downstream of GAL-BD in the pBridge vector to obtain pBD-LcMYB2. The recombinant vectors were introduced into the yeast strain AH109, and positive transformants were selected on SD (−Trp) medium and confirmed by PCR. β-galactosidase activity was assayed according to the Yeast Protocols Handbook (Clontech).

### Arabidopsis transformation

To reveal the biological function of *LcMYB2*, the ORF was fused to the p3301–121 vector (modified from vector pCAMBIA3301 and pBI121, donated by the Shen lab) under the control of the CaMV 35S promoter. The recombinant constructs were transformed into *A. thaliana* by *Agrobacterium tumefaciens* EHA105 using the floral dip method. Positive transgenic Arabidopsis seeds were screened on MS medium supplemented with 20 μg/L glufosinate ammonium and further confirmed by PCR using DNA extracted from the putative positive seedlings. T3 seeds were used for germination assays under different treatments.

### Drought stress tolerance analysis of transgenic seedlings

To reveal the function of *LcMYB2* at the germination stage, T3 seeds of transgenic and wild-type *A. thaliana* were planted on normal solid MS medium and solid MS medium supplemented mannitol (300 m mol/L) or ABA (0.25 μmol/L and 0.5 μmol/L). The Petri dishes were placed in a growth chamber at 22 °C with a 16 h / 8 h, light/dark photoperiod. Plants were photographed, and the germination rate, cotyledon greening rate and root length were and measured. Osmotic and ABA stress tolerance experiments were repeated at least three times.

To test the role of *LcMYB2* at the seedling stage under drought conditions, 4-day seedlings were transplanted into a pot containing vermiculite and turfy soil (2:1, v/v) and grown in a growth chamber at 22 °C with a 16 h / 8 h, light/dark photoperiod for 1 week with sufficient water, then started to natural drought stress without water for the following 42 days. During the drought process, the soil water content was monitored every day in each pot. On the 42nd day, the seedlings were irrigated again, and survival rates were statistically analyzed after 3 days. There are 60 seedlings for drought stress experiment in each line (WT, L1, L2), respectively.

### Measurement of lipid peroxidation and of proline and soluble sugar content

Four-week-old transgenic and WT *A. thaliana* seedlings were irrigated with 300 mmol/L mannitol. The leaves were sampled at 0 h and 9 h after treatment for gene expression analysis. Two days later, the leaves of transgenic and wild-type lines were harvested for physiological measurements. The level of MDAwas determined by a revised method described by Kramer et al. [67]. SOD content was measured with the nitro-blue tetrazolium (NBT) reduction method as previously described [68]. The contents of proline and soluble sugars were determined according to the protocols previously described by Shan et al. [69] and Bailey [70], respectively. Three replicates were carried out for each assay, and the variability was indicated with the standard error (SE).

### Chromatin immunoprecipitation assays

Four-week-old seedlings of sheepgrass were treated with 300 mmol/L mannitol for 8 h and 24 h to induce the expression of *LcMYB2*, after which time, the samples were harvested. Six-week-old seedlings of *A. thaliana* overexpressing *LcMYB2* (L1: line 1, L2: line 2) were tested. All samples were fixed with formaldehyde for CHIP analysis. Antibodies against *LcMYB2* were prepared by Beijing Protein Innovation Co., Ltd. The EpiQuik Plant ChIP kit (Epigentek, Brooklyn, NY) was used for CHIPanalysis. CHIP DNA was detected with universal PCR (40 cycles of 95 °C, 30 S; 68 °C, 30 S) and qPCR (45 cycles of 95 °C, 5 S; 68 °C, 30 S). The method of comparing Ct values was adopted to analyze the qPCR CHIP efficiency.

### Statistical analysis

Analysis of variance (ANOVA) and t-test were used to compare the differences between samples. *** indicates *p*-values < 0.001, ** indicates p-values < 0.01) and * indicates p-values < 0.05.

## Supplementary information

**Additional file 1.** S1 MYB-related transcription factor with unknown function.

**Additional file 2.** S2. Amplification of full-length LcMYB2 and Amplification of LcDREB2 promoter sequence.

**Additional file 3.** S3 Promoter sequence used in CHIP experiment and Possible MYB recognition site.

**Additional file 4.** S4. Primers used in this research.

**Additional file 5.** S5. The root growth experiment.

**Additional file 6.** S6. Pearson correlation analysis between LcMYB2 and other genes.

## Data Availability

Not applicable.

## Abbreviations

ABA: Abscisic acid
AP2/ERF: APETUAP2/Ethylene-Responsive-Element Binding Protein
bZIP: Basic Leucine Zipper
CHIP: Chromatin immunoprecipitation
DREB: Dehydration Responsive Element Binding Protein
LcMYB2: *Leymus chinensis* MYB DNA-binding domain protein 2
LEA: Late-embryogenesis-abundant protein
MS: Murashige-Skoog medium
MYB: MYB DNA-binding domain protein 2
NAC: NAM, ATAF and CUC transcription factor
NCBI: The national center for biotechnology information
ORF: Open reading frame;
P5CS1: △1-Pyrroline-5-carboxylate synthetas
PP2Cs: Plant protein phosphatases
qPCR: Quantitative Real Time PCR
RACE-PCR: Rapid-Amplification of cDNA Ends-PCR
