# Supplementary material for: A MYB-related transcription factor from sheepgrass, LcMYB2, promotes seed germination and root growth under drought stress
Source: BMC Plant Biol. 2019 Dec 18;19:564. doi: 10.1186/s12870-019-2159-2 (PMC6921572; doi:10.1186/s12870-019-2159-2)
Supplement: Supplementary file 1 — Additional file 1. S1 MYB-related transcription factor with unknown function. [file 12870_2019_2159_MOESM1_ESM.pdf]

Additional files

Table S1. MYB and MYB_related transcription factors identification

Gene	RPKM_Control	RPKM_Drought	RPKM_Rewater	type	Score	E-value
contig41717	22.8688947	57.58332	48.41176	MYB	175	3E-57
contig15322	17.73679374	37.78933	30.97388	MYB	258	5E-28
contig38518	10.10650718	24.2016	22.77177	MYB	175	1E-40
contig01062	1.880465132	37.23147	4.064789	MYB	354	1E-37
contig15178	0.2889886	42.63504	12.49348	MYB	368	2E-93
contig44358	39.57482806	5.361926	10.36331	MYB_related	409	8E-31
contig44351	35.69527631	12.70035	344.4308	MYB_related	186	2E-12
contig24103	32.6106677	10.52065	12.07709	MYB_related	338	9E-35
contig00136	23.66852542	6.21474	18.46036	MYB_related	612	2E-11
contig57273	11.77386295	41.7281	26.24555	MYB_related	616	3E-21
contig01121	10.66736584	22.21401	8.573009	MYB_related	491	1E-54
contig37682	9.165365313	63.0881	15.4415	MYB_related	343	3E-159
contig00451	7.822307266	31.72925	9.027471	MYB_related	343	2E-156
contig41859	5.089292411	57.75639	20.94411	MYB_related	387	9E-41
contig24769	1.146439849	25.81515	11.68261	MYB_related	491	2E-39
contig62249	12.28328409	43.91724	21.59513	ERF	370	1E-79

Note: partial data in the Table_S9 from the paper "New Insights on Drought Stress Response by Global Investigation of Gene Expression Changes in Sheepgrass (Leymus chinensis).2016.Frontiers in plant science"
