# Supplementary material for: A MYB-related transcription factor from sheepgrass, LcMYB2, promotes seed germination and root growth under drought stress
Source: BMC Plant Biol. 2019 Dec 18;19:564. doi: 10.1186/s12870-019-2159-2 (PMC6921572; doi:10.1186/s12870-019-2159-2)
Supplement: Supplementary file 2 — Additional file 2. S2. Amplification of full-length LcMYB2 and Amplification of LcDREB2 promoter sequence. [file 12870_2019_2159_MOESM2_ESM.pdf]

1: Amplification of full length LcMYB2

A: 5’-RACE for *LcMYB2*

The first-strand cDNA was synthesized with SMARTer^{TM} RACE cDNA Amplification Kit (Clontech).A gene specific primer and a universal primer was used for 5’-end amplification.

Primers:

5’GSP: 5’- GACAGGCATATAGGGTATTTTC -3’

UPM: 5’- CTAATACGACTCACTATAGGGC**AAGCAGTGGTATCAACGCAGAGT -3’**

5’– CTAATACGACTCACTATAGGGC –3’

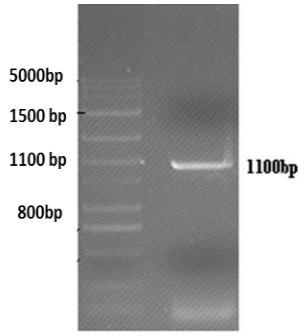

B: Full length amplification of *LcMYB2*

A putative full-length sequence of *LcMYB2* was amplified using the specific pair of primers:

LcMYB2-F: 5’-ATGACGAGGCGGTGCTCGCAC-3’

LcMYB2-R: 5’-GACAGGCATATAGGGTATTTTC-3’

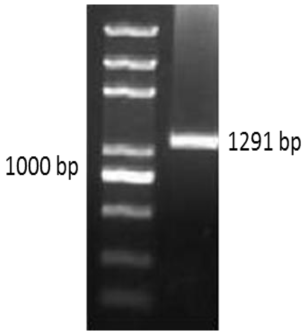

C: OFR region amplification

Primers:

LcMYB2-ORF-F: 5’-ATGACGAGGCGGTGCTCGCAC-3’

LcMYB2-ORF-R: 5’-TCAAACTGCATGGATTGGGCTG-3’

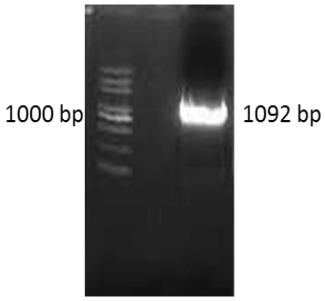

2: Amplification of LcDREB2 promoter sequence

Previous studies revealed that *Leymus chinensis* has closer relationship to *Hordeum vulgare* and *Triticum aestivum* [1], therefore, we search the whole-genome shotgun contigs (WGS) of *Hordeum vulgare* and *Triticum aestivum* with *LcDREB2* (JF754585) and find that partial sequence locating at 5'-end of *LcDREB2* is aligned to gi_413832468 (*Hordeum vulgare*) and gi_439956959 (*Triticum aestivum*) (Fig. 1). Two primers (P1, P4) were designed according to the conserved sequence of *Hordeum vulgare* and *Triticum aestivum* and were used to amplify the putative promoter of *LcDREB2* using total genomic DNA of sheepgrass as template. A fragment about 500bp was amplified and cloned into pMD18-T vector for sequencing (Fig. 2). Three independent clones were sequenced and used to assemble with *LcDREB2* (Fig. 5). According to the assembling sequence (AS1), two primers (P190, P268) were designed to amplify the upstream unknown sequence based the Self-Formed Adaptor PCR [2]. A fragment of about 1000bp was obtained (Fig. 3), ligated into pMD18-T vector, sequenced and assembled with AS1 to get the new sequence (AS2) (Fig. 5). Based on the same method, we acquired another fragment using primers P561 and P784 (Fig. 4). At last, all the sequence were assembled together to form a putative complete *LcDREB2* sequence (Fig. 5). At this time, a primer (F7) picked out in the putative promoter region and another primer (R1821) picked from the coding sequence of *LcDREB2* were used to confirm our previous assembly result. We successfully amplified a 1500bp fragment and sequence analysis revealed that about 200bp of this fragment is the same as the 5'-end of *LcDREB2* (JF754585) (Fig. 6, 7).

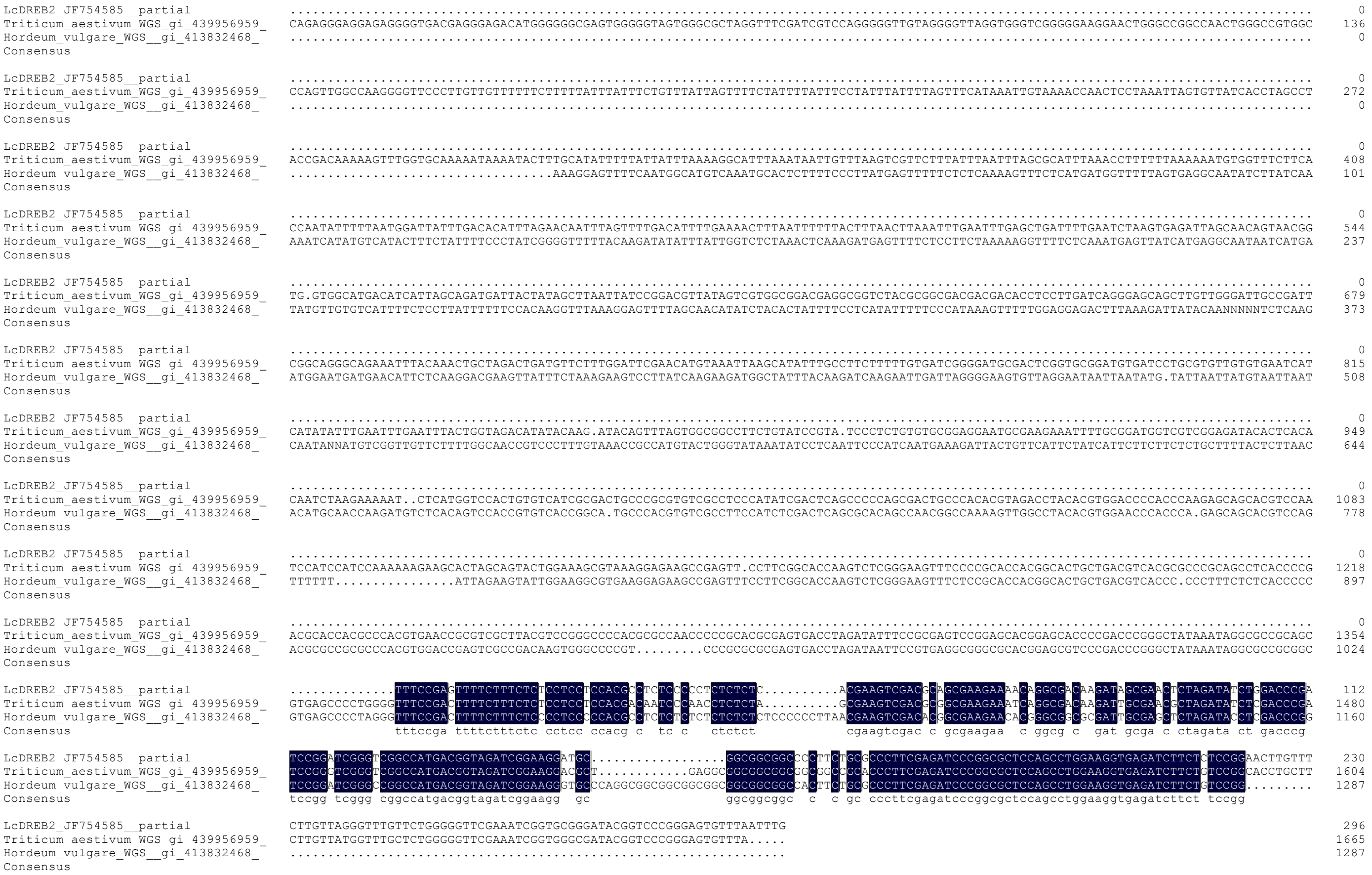

Fig. 1 Sequence alignment of *Leymus chinensis* (*LcDREB2*), *Hordeum vulgare* (gi_413832468) and *Triticum aestivum* (gi_439956959)

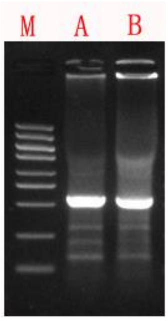

Fig. 2 The single main band on 1% agarose gel showing the products amplified with P1 and P4

M: BMD5000+1.5k ladder, the bands indicate 5, 3, 2, 1.5, 1, 0.75, 0.5, 0.25, 0.1 (kbp) from up to down

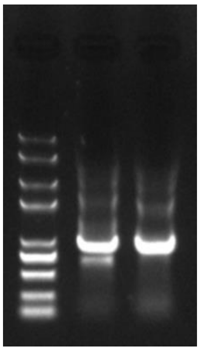

Fig. 3 The single main band on 1% agarose gel showing the products amplified with P190 and P268

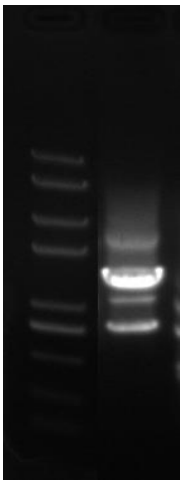

Fig. 4 The single main band on 1% agarose gel showing the products amplified with P561 and P784

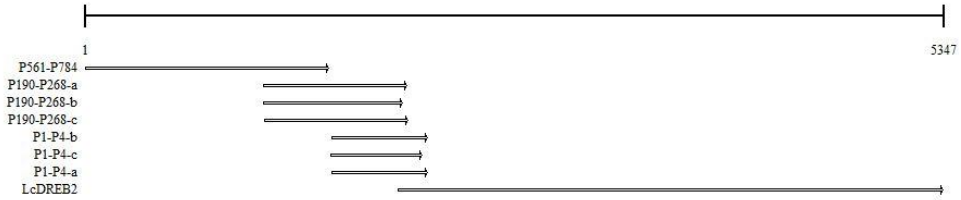

Fig. 5 The assembly result of putative promoter sequence and transcriptional region of *LcDREB2*

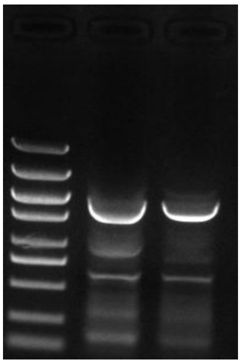

Fig. 6 The single main band on 1% agarose gel showing the products amplified with F7 and R1821

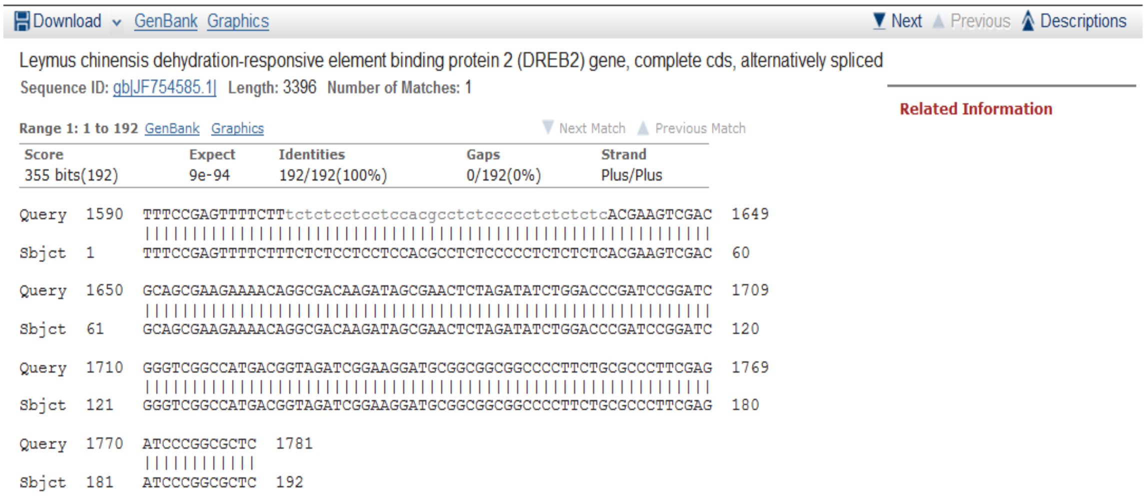

Fig. 7 Result of blastn in NCBI using the sequence amplified with F7 and R1821

Primers used in this experiment

P1：5-CCTTCCGATCTACCGTCAT-3

P4：5-TCTCACAGTCCACCGTGTC-3

P190：5-AGGCGTGGAGGAGGAGAGAAAGA-3

P268：GATACTTCGTGCTCCTGCNNNNNNNNNNTTATC

P561：ATCAGTGGTGGTAAGCGTGGAGTC

P784：TTGCGGAGTTGGAGAAGCNNNNNNNNNNCCAGC

F7：TCTCCAGCTGACGCAACCGCG

R1821：GAGCGCCGGGATCTCGAAGG

Promoter sequence and partial CDS amplified with F7 and R1821 primers

TCTCCAGCTGACGCAACCGCGTGCCTGGCGCGTCAACTGATGGTGCAGAAAACCGTCAGATCCC
TCGGTAGGGGTCCTGGCGTAGCTGGAAGTCTCAGAACGGAGGTCGCACGAGGATTTTACCCAG
GTTCGGGCCTCCGGAGAGTAATACCCTACATCCTGCTCGTGTTTGTTATTCATAGTAGAGGGATAC
CTCGTGCGAGGGGTTTACAATGGTGTGTATGAGATGACTACCGAGAGTTGTTTCTACCGTAGTAG
ATGGGAATGAGAGTTCCCTGGCCTCCCCTTATATACTCGAGGAGGCTAGGGTTTTACTGAGAGAG
GAAACCGATCTAGGGTTGCCACCGCACCAACTTGGAGGTCAAGTTCGCCGCATGGGGTTTATCC
TTATCTTCGGGCTCCCTCTTGTCACTGGGCTTCGCGGGCCCTCCAGCAGGCCTTCTGCGGGGCTC
CCCTCGATGAGCCACCCCCGGTGAACATCAAATTTGTCACCCTACTCGTCGCGAATCCATTCCAT
CATAGTGCACAACCTATTGTTCATTAACAAAGGAGATCGAAAATTCATACGTCATGGCCGGACTA

GAGCGCTCTGCTCTGCGCTGGAGCATCAGGGGCTTGGGGCACGCTGCTGGGGTGGAACCCGGA
GGAGAGGAGGCGGCGGGGCTAGTTGCGGTGGCGCCGACATCGCAGCTACGTGCTAACGTGGGG
TGGCAGTGGCGGCGTAACCCTAGCGCTCGGGCTGGAGAAAGAACGAGAGGAAGAAGTCATGC
GTGACCAGAGATGGCAGCCCCTTGTTTCTCGGCGGTGGCATGCTTTGAAATTTTAGCGAAGTAC
CAAACCGTGCATTTTTTCACTTGGCGGTGGTAGGGGGTGTAATTTGTGTGGACTCCATGAAAATG
GAGAATTCGTGGCGCTGGGAATACCCAGCTTCTCCAACTCCGCCAAAAAATGCACTGAGGCCCA
ATACAGCTTCTCGAATTTAGCTTCGCAGAGCTCAGGCGTTCGGCCCCAGCTTCACACGCGGAGT
TCGCGAAGTGCAGAGCTGGAGAAGGTTCAGAACAAGACCCTTGGGGGACGGATTTGTCACGAA
TGTAGATGCTCTAAGGAAAAGGAAAAGGTGTTGGAGTTGCTCTCGGGACCGGAGAGTGGACTC
CACGCTTGCCACCACTGATCCATGCAACAAAGATGTCTCACAATCCATCTTGTCATCCGACTGCA
CACGTGTCGCCTCCCATATCGACCCAGCCCCCAGCAATTGGCCATACGTGGGCCTACACGTGGA
ACCCACCCAGAGGAGCACGTCCAATCATACAAAAAAAAGGAAGTATACAAAATACTGGAAAGC
GTAAAGGAGAGGCCGAGTTTCCTTCGGCACCAAGTCTCCGGAAGTTTCCCCGCACCACGGCACT
ACTTACGTCACGCGCCCGCAGTCTCACCTCCCACGCACCGCGCCCACGTGGACCGTGTCACTTA
CGACTGGGCCCCACGCGCCAACGTCCGGACGCGAGTGACCTAGATAATTCCGCGGGGCAGGAG
CACGAAGTATCCCGACCCGGGCTATAAATAGGCGACGCGCCGCGTGCCCCTGGGGTTTCCGAGT
TTTCTTTCTCTCCTCCTCCACGCCTCTCCCCCTCTCTCTCACGAAGTCGACGCAGCGAAGAAAAC
AGGCGACAAGATAGCGAACTCTAGATATCTGGACCCGATCCGGATCGGGTCGGCCATGACGGTA
GATCGGAAGGATGCGGCGGCGGCCCCTTCTGCGCCCTTCGAGATCCCGGCGCTC

1. Chen SY, Huang X, Yan XQ, Liang Y, Wang YZ, Li XF, Peng XJ, Ma XY, Zhang LX, Cai YY *et al*: **Transcriptome Analysis in Sheepgrass (Leymus chinensis): A Dominant Perennial Grass of the Eurasian Steppe**. *Plos One* 2013, **8**(7).
2. Wang SM, He J, Cui ZL, Li SP: **Self-formed adaptor PCR: a simple and efficient method for chromosome walking**. *Applied and Environmental Microbiology* 2007, **73**(15):5048-5051.
