# Supplementary material for: A MYB-related transcription factor from sheepgrass, LcMYB2, promotes seed germination and root growth under drought stress
Source: BMC Plant Biol. 2019 Dec 18;19:564. doi: 10.1186/s12870-019-2159-2 (PMC6921572; doi:10.1186/s12870-019-2159-2)
Supplement: Supplementary file 3 — Additional file 3. S3 Promoter sequence used in CHIP experiment and Possible MYB recognition site. [file 12870_2019_2159_MOESM3_ESM.pdf]

1. Promoter sequence

>AtLEA14-Promoter
TTTTGTTTGTATAACCACAACAATTGTTAGTAGATCATATATGAATTGTAATGAAAAATTATATTAGTGACTAGT
GAGAGTTAGTTATTTATATCTTGATTTTTTTTTGTAGTTGGTGGGTTTGGACTCTTTGGTCAAGTCAATCACAT
GCTCTACGTGATTTTACTTGCAGTTCACGCCGGAAACTCTCACGCGTCGACCACGTGACTAGACCTAGTTCAT
AATTTTTATCGTCTGAATGTCAAAAACTCAAATCCTCCTTTTTATAACCGTTTTCTTTGAAAACAAGGTATTTT
CTTAATCTTGTTTCATGTCTTAAGACTAAAGATATTAAAATCTGATTTTGGCTCTACTTATACTAATGTCGTATTA
CAATAACTAAAGTCTCAAACAATATTCTTTAAGCACGCATTGCTGGAAAAATTAATTACAGGGGGGACATAAG
ATCTCAATAATATAGACTGAAGATATCAACTTTTGGCTATTTGGAGTCGTTACATCTACACTTTCAAACAAAAC
CAACGAACCCATTGTTTTTTGGGATCCTACGAATCCCTTGTGTAAACAATAATTAAAGACAGTCCTAAACTAA
TATTCTCTTATCAAGTTTGGAAACATTGTAGCTTTGTGAATATATTATTTTGTCTAAGAAGAACTTTTTGTTTGT
AAATAGTTTTTTTTTTTGGATGTGTTTCTCATGAAATGCAGGTAAGAAACATCTAATGAATTAGAAATAGATTA
TGAATAAAAGAAAATAATTTGTAAAAGAAACACCGAACAAGGAGTTCGTATTCGTATACTAATTGGCCCGCC
AACTACCAACACCGACATATGTGCTTTCGGCAGTTTTGATATCATTCCAAAATTTCAATTATTATTTATCAAATT
TATTTTCTTGGTAAAAGTTTAATTAATTTCAATTATTTGAATGTCTTAATTCACTGATTACAAATTATATATGTAA
TATATACATAACAATTTCGTTCGTGGAGTGTTATTATACTCCTTAAGCGAATCTGTATATTTTAAATAAAATTTTG
ATTTATGCACACGTACATGTTCAATTGAAACGTTTTCCCTAACTATAGATGCCAATAAATAATAAGTTGATTTTT
AAAATGTACAAAACAAAAGGTACCGTTTTGTAAAATTTTGCAACCAAAAAGTACAATCGGCCGTTGAAAATC
GTGGAACCAAACACTAATTAAAGAGAAGTGTGAATATAATCTAGTGTTTGCGGATTTATATTATAAAATTTGTG
TATCACTAAAATGTCAAAGCATGCTTTGACTGGTCAATAAGTCAAATTAACTAACAAACCATGCGAGGCGTA
GGTTTGACTTCTTAACATAAATGCTGACTCAGTTTTTCCATTCCGCAGCTGTCGACTAATTGGACACGCTTCT
TCCAATCCCTTTCCACTAATCATCCATCAACACGTGACATGCCTACAGTGCACGACTTCACAGTGATCATGTC
AAAGTTTTGTTTTTAAGAGAAGAAGATTCACGACTTCACAGTGATCATAGTCCAGTTACAAATTTGAATGGG
CTTATGGCCTGTGGCCCATTTAAGTTTGACCTTAATATCCATCAGAAACCCATACCAATACGGGTCATAAGTGC
ATTGTCCATATAGTGTACCGCAATGCATGGGAGTACTACTACATTCCACCGACGTGCATATGGGGGGACATGG
TCGGTCCATTAATCCCTTGCGCATCTTATTCCTTACGCGAATACTTTTCCCTCTTTTAGTTTTGTATAATACTTTC
CATTTTTTTGGTATTAAATTTATCGTGTGACTATCTAAAAAGGTATTTAATTATCTAGTTTAGCTATAACTGCAA
ACAAAATATCTATTTTCATGAATGATATATTTTTGAAATAAAATATTGTTGTCATGTAACGAAAAAATCAAAAAT
CAAGCAGAAGACACGTATAACCCACGCTTTCACTCTCACTCGTGGAAAACCCTCACACGTACACAAACCATT
ATAAAATTTAATCCATTTACATCGACCG

>AtP5CS1-Promoter
ATTATCGTTTTATGTACCCTTTTTTTTACGTACCAATCAAAATCGCATCTTTCTTTTCAAATATAATTATGTATATA
TACGCATGTGCAGCTGCTGCGTGTCTATGCATACTTGAGTCTAAATAACTATCCCTGACTATGATATGTCGTTG
GTTACGAATGTGATCTTTAGTTAAAATAACAAGAATAATATCACACAGACAAAAACAAATAAGTGCATATTAT
TTCAACAAGATTGGAGTGGGTGGGCCTAAAGGCTTAAAAAAATAGAGCACATGCACAGAGGACCATTGATT
CCCCAGAGACAATCAGACATCTGAGACCCTAATCGCATCAAGCCGCGTGCCCTTCTTCCATTAACTAATTTTG
TGTGTTTGTTTTGGCTTAAACCTGAGAATTACATTGATTACTTTATTTGTTTCATTTTCTCCATGAAGGAGATAA
AAAGAGTAAAAATTAGAGATTGATGAAAACTGAAAAAGAAACTGAAGTCGGTAAGATAGGTGTTGGAACTT
GGAATAATGGCTTGGCTTTGAACAAAACGCATGCACCATTCATTGCCTTCAAGTTTTTTGCAATTAGCTTTGT
TTTTGTTTTTGTTTTTGTTTTTGGGAGAGGTTCTAATGACCAAGAATCAAGAGCGTTGTCTAAAATCTAAACC
ATATGATACGGTTTTTAATATTCTCATGCATTAAAATGTACTATTTCTATATATGATCTTATATACCCAACATCTTG
GAATTAATAGTTTGATTCGTTATCATTTGAAGAAGCTCTCAACAGCTTCAAAAAGCGAAATGTAGCATCATGA
AGCGGTATCCAATTTCAAGAAGCTACCAGTAGCTTGTGGAAGTTTTCAAGAAGTTTTCCGGAAGATCCAACG

ATTGTGGAAGCCCCTCATAGTTTTTGGAAGTTTTCAATGATATTAGCAGCGTTGAGCGTGGCATGGCTAGACA
ATGTAAGAGATTTGATTTGCAACACATTTGATGTATTTTTTTACTTTTGAGTTACAATTGTAATGTATTATTGATT
TTGCCCAGTTATGATTTATAAACCCTACAATTTAGTATCAAAGTTTTTATTTAAAATTCTGAATCTGACATTAAT
GATATCTGGCTCATTTACAGAGCCAATGAGATGGATGATGTTCGAAACTGGATTGGCCATTATTTATCTTTTTT
TTATCTGGAGAATCTCGAGGTTGGCACAAACATTATCATATTAGCCTTTAGAAATTGGATTGGCTAATCACACA
TTTATATATATTCTTACCAAAATAAATCACCTCTCCCGTAATTGAAAAATATCTAAATACTGTAAGTCTGAAAAA
ATTCACAAGGGTTCGAAGAAAGAAGGAAATATCTAAGCATCATTAATAAACTATCTGTAACCTGAGGGAAAA
TCATTTCATGTTGAAATATGTGGATTTGGAAGTTTTATAATCTATCTGAATTTGTGAAATTTGATAACAAGTAA
GATTTGTTTCTTAACACAAATCTAAAATTTGTTTTCTAATTAGGTTTGAGAGAGAGAGAGAAAGAAACGCTTT
GTATGATACACATCTAGGCTATGAATGAAGGCAGCGGACAAAGCGGTCTAATTTGTCTGCGGTTTAGTCCATC
TCATTTTTGGGGTGGACAATAAACCGCTGCGGACCAAGTTTATTTGTATGTAAAAACGGTCCGCAGATGGTC
CGCAACGATTTTCTTCTATTTTTTTAAGTCCAGACCACTGCGGACTATAATTGATGAATGATAAATAAAAAACG
GTCTGATCCGTTGACGGTTTTGTCCGCCCCAACCGCCATAACCATTCAAACCCCTAATTATTTCATCAGATAAC
ATTATACACTAATAATCATTGCACTCAAATATGTCACACAATCATATAATAAAATAATAACAATGATTAAAATGA
AAAAATTGTTGTGGCGCCGCATAAAATAGAAATCGTGAGAGACGACGTCATCTAAAAATTGCCTTGCTGTCC
ACTTTTCACTTTGTCCTCTCTTCTCATCTCCGTTCA

>AtDREB2A-Promoter
TTTTCAACAAATCATCTCGTCTTGCCTCCAACTGTCGTACAGTTTTCTTTAGAGCCGCTACATGATCTACGAG
GTTGCGGAAGCGATTTTGGTTCCTGCTAAGGCAGCTTGTAAGGTTATTAACAGCCTGGTCACAGGATATTGCC
ACAGAGAAGCAGGCTCCCATCTCTCATAGATTCTCAAATTCCTACCAGAGAGATTAATATACAAATTCCTTTC
CTAATGTTTTAGTTTATTAACTGAGGTGTGTGCACGATCATTCGGGTTGACCACCAATCTTGGCATCCAGTCAT
CTACGACTCGATCAAATATTTATGTTATTTTAAAAATGAACTGTAACCAAGATAACATTTTTTTCATAAGGTCA
ACGACCCTTGGGATGTTAACAGAGCCAAGTTTAGTGAGCACTATGTGCTCGTTTTTTCTTAACACAATTCTAA
CGTGGTTTGGCCAAGCCAAGTCAACATTTTACTTACATCTCGTTTTCCAATAATGGTTTTGTAATCGATAACCT
AAATCATTGTAATGAATGCGTTCCTCCCTATCGATCCTAGGCCTTAGACAATGCTGAATGATTCATAGCCACGC
GAATAACCTATTCATACTAATATGGAAAGAAAGAAGCCAAACTTACAGAGCTCTTCTCACGGTCGTCGAAGA
AAAGAGAGTCTACAGTCAGCAACAAAATTAGTGTTGCCATCATGGCATCATCTTGCAGCTTTTTCCGCACAA
ATAATTTATCATCCAAATGTTGTTCACTAAACCAAAAACAAACAAGCATTACAGCGAAGAAACCAACTCGTA
GATACAGATTTCCAAATTCATGCCTTATTTAGACCAATAAAAACTGAAATTTCTCTTCAGCGAAAAAAAAAAC
AAACAAACTGAAATTTCATATAGATCCAGAAGATAGAAACTTGTAGGCTCAATCGACTAGACTAGAAGATGC
TCACCCGATCGTGCTTGAGATAGCGAGATAGTAGCAACACCGACGGTAGAAATAAAATGGACGACACCCATC
CAATGGGCTAATTTAGATTAACGGGCTTTAAGGGTTTGATAATGGATGTTAATTAACTGAGGCACATGGGATT
GTATCACGTAGGCAATGGGTTTGATAATGGATGTTAAGTAACTAAGGCCCATGAGGTTGAAGTACGTAGGCAA
TTGCGTGAGCTTACGTTAGCGATGCCGTTAGAGACACGTAGTGGATGAAGTGGCTTTGGTTAGCAAAGGACA
CATGAGGCACATGCAAAGGCTATAAATGACTGCTGCTTTGCTACAACTTGCGATTCCCAAATTTTATAAGGTA
ATGGACCCTATCACCTCTGCTCGAAGCTAAGCCACCCAAGTTTGAGCTTCACCATTTGACACGTCTCTAGCTA
ATACTTAATGCTTAAACTTTAAACTAATACTTCATGTTTAAGACATTTCTGGCTGACACATTTATGAATTCGCTC
TATGTCGTACGTACACCGGAAACCTTTAATTTTACAATATTAACCGTGATCTTTTTAAAAATATCTATATAAACG
ATAAGTATCTCATCAAAACAAAATTGAATAATGTGCACGTTTGTAAAAACTAGAAAAACAGGCAATAAACAT
CATCATCCAATTACACATCTAGTAAGTGTGATGCAGTGGCAAACTGGCAATAAACCAAGAAAAATCGAGAAA
GAGCAGATGAGACAGTGTTGTGTTGTCAGGGTTAATAAAAAAAAAATGAAGATATTTTAAAATTTCATAATAT
TTAAATAATGAAGTAGTTTTATGATCTTATCCATAAATCAATTTTAAAAAGGTTTAAACTTTACTTTTCCGTATC
AACAGCGTGTTTCGAGAAGATTCGGGAGGACACTCGTCGAACGGAAAAGTCGTCTAAGCCTTTATGTTCGA
ATCAAAAACTGACACGTAACCTTGCTCTCAAACAGAAAAATAAAATAATGTTAGAAAAATCTAGAGAAGGCT

ATAAATACTCCGTAGATACTTTGTCTTCCTT

>LcDREB2-Promoter
TCTCCAGCTGACGCAACCGCGTGCCTGGCGCGTCAACTGATGGTGCAGAAAACCGTCAGATCCCTCGGTAG
GGGTCCTGGCGTAGCTGGAAGTCTCAGAACGGAGGTCGCACGAGGATTTTACCCAGGTTCGGGCCTCCGG
AGAGTAATACCCTACATCCTGCTCGTGTTTGTTATTCATAGTAGAGGGATACCTCGTGCGAGGGGTTTACAAT
GGTGTGTATGAGATGACTACCGAGAGTTGTTTCTACCGTAGTAGATGGGAATGAGAGTTCCCTGGCCTCCCC
TTATATACTCGAGGAGGCTAGGGTTTTACTGAGAGAGGAAACCGATCTAGGGTTGCCACCGCACCAACTTG
GAGGTCAAGTTCGCCGCATGGGGTTTATCCTTATCTTCGGGCTCCCTCTTGTCACTGGGCTTCGCGGGCCCT
CCAGCAGGCCTTCTGCGGGGCTCCCCTCGATGAGCCACCCCCGGTGAACATCAAATTTGTCACCCTACTCGT
CGCGAATCCATTCCATCATAGTGCACAACCTATTGTTCATTAACAAAGGAGATCGAAAATTCATACGTCATGG
CCGGACTAGAGCGCTCTGCTCTGCGCTGGAGCATCAGGGGCTTGGGGCACGCTGCTGGGGTGGAACCCGG
AGGAGAGGAGGCGGCGGGGCTAGTTGCGGTGGCGCCGACATCGCAGCTACGTGCTAACGTGGGGTGGCA
GTGGCGGCGTAACCCTAGCGCTCGGGCTGGAGAAAGAACGAGAGGAAGAAGTCATGCGTGACCAGAGAT
GGCAGCCCCTTGTTTCTCGGCGGTGGCATGCTTTGAAATTTTAGCGAAGTACCAAACCGTGCATTTTTTCAC
TTGGCGGTGGTAGGGGGTGTAATTTGTGTGGACTCCATGAAAATGGAGAATTCGTGGCGCTGGGAATACCC
AGCTTCTCCAACTCCGCCAAAAAATGCACTGAGGCCCAATACAGCTTCTCGAATTTAGCTTCGCAGAGCTCA
GGCGTTCGGCCCCAGCTTCACACGCGGAGTTCGCGAAGTGCAGAGCTGGAGAAGGTTCAGAACAAGACC
CTTGGGGGACGGATTTGTCACGAATGTAGATGCTCTAAGGAAAAGGAAAAGGTGTTGGAGTTGCTCTCGG
GACCGGAGAGTGGACTCCACGCTTGCCACCACTGATCCATGCAACAAAGATGTCTCACAATCCATCTTGTCA
TCCGACTGCACACGTGTCGCCTCCCATATCGACCCAGCCCCCAGCAATTGGCCATACGTGGGCCTACACGTG
GAACCCACCCAGAGGAGCACGTCCAATCATACAAAAAAAAGGAAGTATACAAAATACTGGAAAGCGTAAAG
GAGAGGCCGAGTTTCCTTCGGCACCAAGTCTCCGGAAGTTTCCCCGCACCACGGCACTACTTACGTCACGC
GCCCGCAGTCTCACCTCCCACGCACCGCGCCCACGTGGACCGTGTCACTTACGACTGGGCCCCACGCGCCA
ACGTCCGGACGCGAGTGACCTAGATAATTCCGCGGGGCAGGAGCACGAAGTATCCCGACCCGGGCTATAAA
TAGGCGACGCGCCGCGTGCCCCTGGGG

2. Possible MYB recognition site

AtLEA14-P   TAACTG -185
AtLEA14-P   TAACCA -2014
AtLEA14-P   AAACCA -1510
AtLEA14-P   AAACCA -709
AtLEA14-P   AAACCA -37
AtP5CS1-P   TAACCA -220
AtP5CS1-P   AAACCA -1370
AtDREB2A-P CAACTG -1974
AtDREB2A-P TAACGG -963
AtDREB2A-P TAACTG -1766
AtDREB2A-P TAACTG -928
AtDREB2A-P TAACCA -1666
AtDREB2A-P AAACCA -1242
AtDREB2A-P AAACCA -1211
AtDREB2A-P AAACCA -344
LcDREB2-P   CAACTG -1559
