# Supplementary material for: A MYB-related transcription factor from sheepgrass, LcMYB2, promotes seed germination and root growth under drought stress
Source: BMC Plant Biol. 2019 Dec 18;19:564. doi: 10.1186/s12870-019-2159-2 (PMC6921572; doi:10.1186/s12870-019-2159-2)
Supplement: Supplementary file 4 — Additional file 4. S4. Primers used in this research. [file 12870_2019_2159_MOESM4_ESM.pdf]

Primers used in this study

Name	Sequence(5'-3')	Purpose
GSP-5'RACE	GACAGGCATATAGGGTATTTTC	5' RACE
LcMYB2-F	ATGACGAGGCGGTGCTCGCAC	Full length amplification
LcMYB2-R	GACAGGCATATAGGGTATTTTC	Full length amplification
LcMYB2-ORF-F:	ATGACGAGGCGGTGCTCGCAC	open reading frame amplification
LcMYB2-ORF-R:	TCAAACTGCATGGATTGGGCTG	open reading frame amplification
Semi-qPCR-LcMYB2-F	CGGCGTTCTACCCATCAT	Semiquantitative PCR
Semi-qPCR-LcMYB2-R	TGTTGTTTCCCTGACTTAGGTC	Semiquantitative PCR
Semi-qPCR-LcActin-F	GCACCCTGTGTTGCTCACT	Semiquantitative PCR
Semi-qPCR-LcActin-R	TACCTTGATCTTCATGCTGCTC	Semiquantitative PCR
qPCR-LcMYB2-F	GGGCCTTCCTAGTCAAGTCTTACAGA	Quantitative PCR
qPCR-LcMYB2-R	GTACAAGCGCAGGAGCTTACGC	Quantitative PCR
qPCR-LcActin-F	GTGCTTTCCCTCTATGCAAGTGGT	Quantitative PCR
qPCR-LcActin-R	CTGTTCTTGGCAGTCTCCAGCTC	Quantitative PCR
pCAMBIA1302-LcMYB2-GFP-F	CGACTAGTATGACGAGGCGGTGCTCGCAC	Subcellular location
pCAMBIA1302-LcMYB2-GFP-R	CCCATGGAACTGCATGGATTGGGCTG	Subcellular location
pBridge-LcMYB2-F	GGAATTCATGACGAGGCGGTGCTCGCACT	Transcription activity assay
pBridge-LcMYB2-R	CGGGATCCTCAAACTGCATGGATTGGGCT	Transcription activity assay
p3301-121-LcMYB2-F	CGGGATCCATGACGAGGCGGTGCTCGCAC	Genetic transformation
p3301-121-LcMYB2-R	CGAGCTCTCAAACTGCATGGATTGGGCTG	Genetic transformation
qPCR-AtACTIN-F	TGCTGACCGTATGAGCAAAG	Quantitative PCR
qPCR-AtACTIN-R	GATTGATCCTCCGATCCAGA	Quantitative PCR
qPCR-AtMYB2-F	AACGTCTTCGAATTCTCCGGCTGA	Quantitative PCR
qPCR-AtMYB2-R	ATCGTTGAACTCTCCGAAACCCGT	Quantitative PCR
qPCR-AtP5CS1-F	TAGCACCCGAAGAGCCCCAT	Quantitative PCR
qPCR-AtP5CS1-R	TTTCAGTTCCAACGCCAGTAGA	Quantitative PCR
qPCR-AtLEA14-F	GATTTCTTCTGATCGACAAAACCTA	Quantitative PCR
qPCR-AtLEA14-R	AGCAAACCCAACTTATTACATTACG	Quantitative PCR
qPCR-AtDREB2A-F	AAACCTGTCAGCAACAACAGCAGG	Quantitative PCR
qPCR-AtDREB2A-R	TTAAGCCTGCAAACACATCGTCGC	Quantitative PCR
qPCR-CHIP-AtP5CS1-F1	TCCAGACCACTGCGGACTATAATTGATGAA	CHIP-enrichment
qPCR-CHIP-AtP5CS1-R1	GCGGTTGGGGCGGACAAAAC	CHIP-enrichment
qPCR-CHIP-AtP5CS1-F2	ATCTAGGCTATGAATGAAGGCAGCGGACAA	CHIP-enrichment
qPCR-CHIP-AtP5CS1-R2	GTTGCGGACCATCTGCGGACC	CHIP-enrichment
qPCR-CHIP-AtP5CS1-F3	AATAGAGCACATGCACAGAGGACCATTGAT	CHIP-enrichment
qPCR-CHIP-AtP5CS1-R3	CGGCTTGATGCGATTAGGGTCTCAGATG	CHIP-enrichment
qPCR-AtLEA14-CHIP-F1	GCCTGTGGCCCATTTAAGTTTGACCTT	CHIP-enrichment
qPCR-AtLEA14-CHIP-R1	CCATATGCACGTCGGTGGAATGTAGTAGT	CHIP-enrichment
qPCR-AtLEA14-CHIP-F2	ACCATGCGAGGCGTAGGTTTGACTT	CHIP-enrichment
qPCR-AtLEA14-CHIP-R2	AGTGGAAAGGGATTGGAAGAAGCGTGTC	CHIP-enrichment
qPCR-AtLEA14-CHIP-F3	TTTGTAGTTGGTGGGTTTGGACTCTTTGGT	CHIP-enrichment
qPCR-AtLEA14-CHIP-R3	GCGTGAGAGTTTCCGGCGTGAA	CHIP-enrichment

qPCR-AtDREB2A-CHIP-F1	CGAGAAAGAGCAGATGAGACAGTGTTGTGT	CHIP-enrichment
qPCR-AtDREB2A-CHIP-R1	CGACGAGTGTCCTCCCGAATCTTCT	CHIP-enrichment
qPCR-AtDREB2A-CHIP-F2	CGATGCCGTTAGAGACACGTAGTGGATGAA	CHIP-enrichment
qPCR-AtDREB2A-CHIP-R2	TCGCAAGTTGTAGCAAAGCAGCAGTCA	CHIP-enrichment
qPCR-AtDREB2A-CHIP-F3	AGAGCCGCTACATGATCTACGAGGTT	CHIP-enrichment
qPCR-AtDREB2A-CHIP-R3	GCTTCTCTGTGGCAATATCCTGTGACC	CHIP-enrichment
qPCR-LcDREB2-CHIP-F1	GCGAAGTGCAGAGCTGGAGAAGGT	CHIP-enrichment
qPCR-LcDREB2-CHIP-R1	ATCAGTGGTGGCAAGCGTGGAGTC	CHIP-enrichment
qPCR-LcDREB2-CHIP-F2	CGGGCTGGAGAAAGAACGAGAGGAAGAAG	CHIP-enrichment
qPCR-LcDREB2-CHIP-R2	ATGCCACCGCCGAGAAACAAGGG	CHIP-enrichment
qPCR-LcDREB2-CHIP-F3	TCCTGGCGTAGCTGGAAGTCTCA	CHIP-enrichment
qPCR-LcDREB2-CHIP-R3	CGGTAGAAACAACTCTCGGTAGTCATCTCA	CHIP-enrichment
