# Supplementary material for: A MYB-related transcription factor from sheepgrass, LcMYB2, promotes seed germination and root growth under drought stress
Source: BMC Plant Biol. 2019 Dec 18;19:564. doi: 10.1186/s12870-019-2159-2 (PMC6921572; doi:10.1186/s12870-019-2159-2)
Supplement: Supplementary file 5 — Additional file 5. S5. The root growth experiment. [file 12870_2019_2159_MOESM5_ESM.pdf]

L1 WT L2

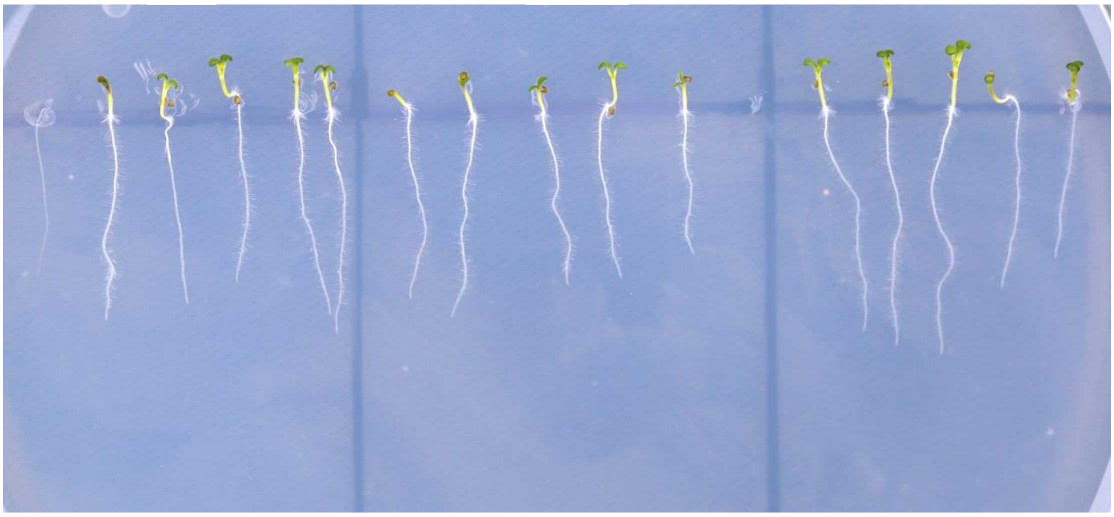

Figure A：The root growth experiment directly on MS medium. The left in the panel is L1, the middle is WT, and the right is L2.

L1 WT L2

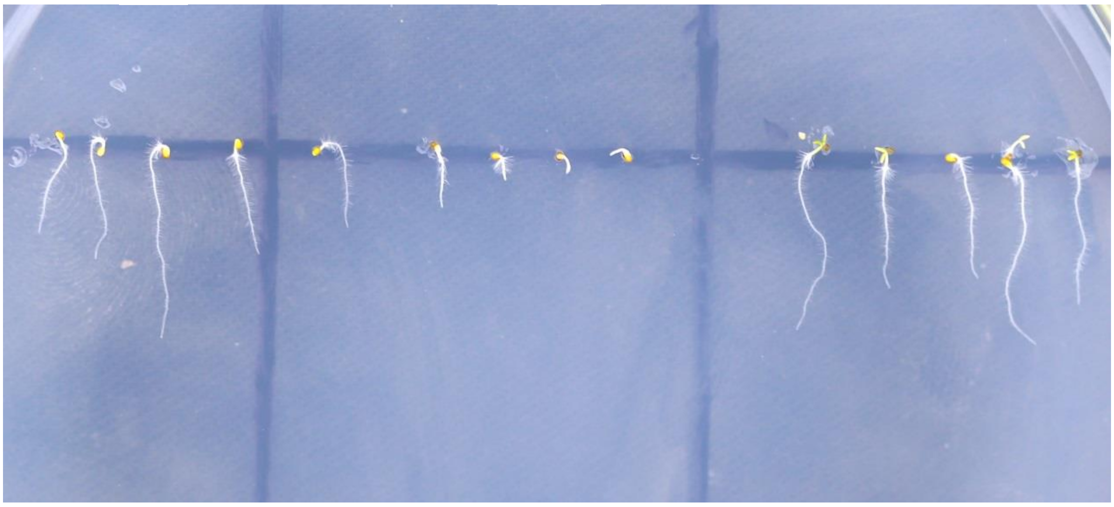

Figure B: The root growth experiment directly on MS with 300mM mannitol medium. The left in the panel is L1, the middle is WT, and the right is L2.
