# Supplementary material for: A MYB-related transcription factor from sheepgrass, LcMYB2, promotes seed germination and root growth under drought stress
Source: BMC Plant Biol. 2019 Dec 18;19:564. doi: 10.1186/s12870-019-2159-2 (PMC6921572; doi:10.1186/s12870-019-2159-2)
Supplement: Supplementary file 6 — Additional file 6. S6. Pearson correlation analysis between LcMYB2 and other genes. [file 12870_2019_2159_MOESM6_ESM.pdf]

Pearson correlation analysis between *LcMYB2* and other genes

GeneID	Annotation	contig41859	
		r	pvalue
contig41859	LcMYB2	1	0
contig15297	GA2OX8	0. 99990	0. 00897
contig15178	MYB39	0. 99990	0. 00893
contig44866	Peroxidase 56	0. 99997	0. 00473
contig62249	LcDREB2c	0. 99997	0. 00465
contig37697	SnRK	0. 99991	0. 00830
2-GH8N3EB02J6KME	LEA	0. 99999	0. 00256
